# Needing to shout to be heard? Caregiver under‐responsivity and disconnection between vocal signaling and autonomic arousal in infants from chaotic households

**DOI:** 10.1111/cdev.14183

**Published:** 2024-11-08

**Authors:** S. V. Wass, C. S. Smith, F. U. Mirza, E. M. G. Greenwood, L. Goupil

**Affiliations:** ^1^ Department of Psychology University of East London Stratford UK; ^2^ Institute of Psychiatry, Psychology & Neuroscience King's College London London UK; ^3^ LPNC, Université Grenoble Alpes/CNRS Grenoble France

## Abstract

Children raised in chaotic households show affect dysregulation during later childhood. To understand why, we took day‐long home recordings using microphones and autonomic monitors from 74 12‐month‐old infant–caregiver dyads (40% male, 60% white, data collected between 2018 and 2021). Caregivers in low‐Confusion Hubbub And Order Scale (chaos) households responded to negative affect infant vocalizations by changing their own arousal and vocalizing in response; but high‐chaos caregivers did not, whereas infants in low‐chaos households consistently produced clusters of negative vocalizations around peaks in their own arousal, high‐chaos infants did not. Their negative vocalizations were less tied to their own underlying arousal. Our data indicate that, in chaotic households, both communicating and responding are atypical: infants are not expressing their levels of arousal, and caregivers are under‐responsive to their infants' behavioral signals.

**Abbreviations**
chaos: Confusion Hubbub And Order Scale
ECG: electrocardiogram

## INTRODUCTION

A lack of family and weekly routines, high noise levels, disorganization, and crowding are all part of household Confusion Hubbub And Order Scale (chaos) (Evans & Wachs, 2010; Matheny et al., 1995). Chaos is here defined in accordance with the 15‐item questionnaire used, where higher scores represent more chaotic, disorganized, hurried characteristics of the home environment, found to overlap with trained observers' as well as parent‐reported observations of noise and crowding (Matheny et al., 1995). Higher levels of household chaos have been related to developmental delays across a range of areas including cognitive and academic outcomes (Deater‐Deckard et al., 2009; Shamama‐tus‐Sabah et al., 2011), language acquisition (Martin et al., 2011), mental and physical health outcomes (Coldwell et al., 2006; Marsh et al., 2020; Mills‐Koonce et al., 2016), and affect dysregulation (Bobbitt & Gershoff, 2016; Coldwell et al., 2006; Deater‐Deckard et al., 2009; Martin et al., 2011; Miller et al., 2017) (see Marsh et al., 2020 for a review). Higher levels of household chaos are also known to relate to less responsive and more intrusive caregiving styles (Andeweg et al., 2020; Deater‐Deckard et al., 2012; Dumas et al., 2005; Geeraerts et al., 2021; Nelson et al., 2009; Valiente et al., 2007; Zvara et al., 2020). Vernon‐Feagans and colleagues examined the relationship between household chaos, early executive function and behavioral regulation, and caregiver responsivity in a longitudinal study of 1145 infants. Household chaos and caregiver responsivity were rated by trained researchers at each of the 5 home visits, and executive function tests were administered at older ages. The results suggested that the relationship between household chaos and executive function was mediated by caregiver responsivity (Vernon‐Feagans et al., 2016) (see also Geeraerts et al., 2020; Song et al., 2018).

The adverse effects of chaos on caregivers and children may operate through disruption to routines as well as subjective feelings of hecticness and stress (Fiese & Winter, 2010). Chaotic households have been found to lack consistent routines, such as at sleep or mealtimes, where family members may spend more structured face‐to‐face time together (Appelhans et al., 2014), and also be more likely to involve increased screen use in preschool‐aged children (Emond et al., 2018), potentially pointing to a lack of parental monitoring or willingness to entertain children through media rather than interaction. Stressed, fatigued caregivers may also find it more challenging to find the time and/or energy to engage in sustained interaction with their children, with these interactions less likely to be positive (Nelson et al., 2009; Valiente et al., 2007; Zvara et al., 2020). The key to appreciating how caregiver under‐responsivity might lead to child affect dysregulation is likely to lie in understanding the dynamic bi‐directional influences between children and the caregivers.

Infants are thought to learn self‐regulation by experiencing repeated cycles of co‐regulation with their caregiver; over time, the infant internalizes the expectation of the caregiver's soothing response and through this learns self‐regulation (Bronfenbrenner, 1977; Ham & Tronick, 2009; Kopp, 1982; Olson & Lunkenheimer, 2009; Wass et al., 2024). In home settings, for example, caregivers respond to their infants' distress by upregulating their own arousal state to match their child's, leading to short‐term increases in arousal synchrony; greater caregiver responsivity to infant stress associates with faster infant quieting (Wass et al., 2019, 2022). In lab settings, studies have similarly shown that the recovery period of the infant following a “still face” procedure is dependent on the response of the caregiver during the “reunion” phase (Enlow et al., 2014; Feldman, Gordon, et al., 2010; Provenzi et al., 2015). These studies have, however, concentrated on understanding how caregiver under‐responsivity is driven by the caregiver adapting (or not) to their child. Less research has investigated bi‐directional links, examining simultaneously the role of the child as a “sender” in these exchanges.

To understand how an infant may communicate their distress differentially considering the environment, it is important to consider how an infant's stress response itself may be affected by the environment. Infant stress reactivity has been postulated to show a “biological sensitivity” to the caregiving context, with a U‐shaped relation to levels of adversity, whereby exposure to acutely stressful or extremely supportive environments confer a benefit to highly reactive profiles, up‐regulating over development the stress response in these environments (Boyce & Ellis, 2005). Indeed, studies have demonstrated the benefits of moderate levels of negative reactivity in sensitive caregiving environments, linking moderate levels of negative fussing and variability in cortisol in infants to better later self‐regulation and executive function in toddlerhood and early childhood respectively (Blair & Berry, 2017; Geeraerts et al., 2020). The effects of an adverse caregiving environment on stress reactivity are nuanced. Examining the impact of exposure to childhood adversity on diurnal cortisol levels, Gustafsson et al. (2010) found increased cortisol levels among moderately exposed children but that children most exposed exhibited low levels, suggesting that an initially upregulated stress reactivity in response to adverse circumstances eventually becomes blunted in those exposed to chronic stressors. Early‐life adversity leading to blunted cardiovascular and cortisol reactivity has been demonstrated by a meta‐analysis of 83 studies examining the association (Brindle et al., 2022). Such findings fit allostatic load theory, whereby allostasis is engaged as a response to acute stress but becomes overworked and eventually suppressed due to chronic engagement or repeated failure to recover from the stress response (McEwen, 1998). Although the stressors of chaotic homes may not be directly comparable to the more severe adversity examined in the literature above (though chaos has been linked to increased cortisol levels in low‐income households, Brown et al., 2019), the plasticity of initial allostatic setpoints in the stress response system highlights the importance of unpicking the environmental influences on infants' stress response as well as the signaling of distress.

From birth, infants typically express signs of heightened arousal and distress through cries and facial expressions. It is thought that, initially, infants mostly express these communicative displays automatically—that is, directly as a function of autonomic arousal and physiological responses to their environment (Craig, 1992; Zeskind, 2013). This predicts that, early on, physiological states of arousal and (facial and vocal) displays of stress/distress should typically align. For instance, in the absence of dysregulation, infants should mostly cry when they are aroused, and not otherwise (Zeskind, 2013). Over time, infants develop the ability to use displays flexibly and intentionally (Feldman, 2007; Matthews, 2020; Miller & Sperry, 1987). The onset of such intentional communication can for instance be seen in gaze‐coordinated vocalizations—where children vocalize while looking toward their caregivers, or alternate their gaze between their caregiver and a situation or object (Bates, 2014; Donnellan et al., 2020; Schieffelin, 2016). Vocal and facial communication then complexifies and diversifies according to specific display rules that are culture‐specific and that are transmitted to children by caregivers, sometimes in very explicit ways (Miller & Sperry, 1987).

Early on, caregiver responsivity is thought to be crucial for the transition toward intentional communication (Albert et al., 2018; Locke, 2006; Matthews, 2020). Infants' dynamic behaviors, such as crying and smiling, influence their caregivers' behaviors (Feldman, 2007), and negative vocalizations including cries elicit greater (Tronick, 2007; Wass et al., 2019, 2022) and faster (Yoo et al., 2018) responses from caregivers, while vocalizations that are more speech‐like prompt more contingent and non‐overlapping responses (Albert et al., 2018; Yoo et al., 2018). This signifies that caregivers are sensitive to variations in the prosodic and phonetic characteristics of their infants' vocalizations, and they interpret them as serving various functions (e.g., cries are typically perceived as requiring a more urgent response, whereas babbling sounds are interpreted as precursors of speech that should be embedded in a turn‐taking, conversation‐like structure). Yet it remains unclear whether, and if so how, infants' vocal and facial displays affect caregiver responsivity differently as a function of their arousal level, and how these relationships may vary as a function of contextual factors such as household chaos.

Few previous studies have investigated simultaneously the relationship between infants' displays, their autonomic arousal, and caregiver responsivity. One recent study looked at vocalization and arousal co‐fluctuations across the day in naturalistic home environments; it found that negative affect vocalizations (including cries, which accounted for 98% of negative vocalizations), occurred most frequently when the infant was highly aroused and with lower arousal stability (Wass et al., 2022) (see also McFarland, 2001; McFarland et al., 2020; Wilder, 1974). These types of vocalizations also elicited the highest frequency of caregiver contingent responses when compared to more positive vocalizations. High intensity infant vocalizations also coincided with greater infant arousal changes, followed by a period of arousal stability across the dyad. This confirms that cries—as well as being particularly salient for caregivers—are an important tool for establishing co‐regulation within the dyad (Wass et al., 2019, 2022). It thus seems important to understand what happens in noisy, chaotic environments that can bidirectionally influence how caregiver responsivity relates to infants' expressions of distress, potentially in turn affecting the development of infants' self‐regulatory skills.

To address this, in study 1 we used miniature wearable microphones and autonomic monitors to obtain day‐long recordings in home settings from a cohort of *N* = 74 12‐month‐old infants across the South‐East UK. We examined how autonomic arousal, in infant and caregiver, changed relative to naturally occurring infant vocalizations during the day. Participating caregivers also attended a lab visit, where an adapted still face procedure (Ham & Tronick, 2009), a widely used test of emotion regulation in infancy, was recorded (study 2). We examined changes in autonomic arousal and facial affect both during the still face procedure, and afterward.

For study 1, trained, blinded coders manually coded all infant vocalizations across two dimensions: first, vocal affect, ranging from negative (fussy and difficult) to positive (happy and engaged); and second, vocal intensity, reflecting the intensity with which the affect was expressed. Because of limited storage space our microphones recorded a 5‐second sample every minute and our analyses examined only large‐scale arousal changes in infant and caregiver arousal and vocal behaviors during the 10 min before and after each vocalization. It is therefore important to note that, in characterizing caregiver responsiveness, we follow on from previous research (e.g. Smith et al., 2023) in documenting coarse‐grained contingency—i.e., changes in caregiver arousal and vocal behavior in the period of 0–10 min following infant distress, rather than fine‐grained contingency—i.e., responses of the caregiver in the period 0–10 s following infant distress (see e.g., Tamis‐Lemonda et al., 2014). Available evidence suggest that temporally fine‐grained and coarse‐grained caregiver contingency are likely to be inter‐related (Wass et al., 2024), although we did not address that explicitly in this study. It is also noteworthy that our recordings did not capture all vocalizations that occurred—although, because the sampling happened at random, the presence of undetected vocalizations ought to only have weakened any patterns of event‐related change that we did observe. Separate analyses (shown in Section S1.10) confirm that the temporal patterning of vocalizations in our data was preserved despite this sparse coding approach.

We hypothesized that higher levels of household chaos should associate with reduced caregiver responsivity to their infant's vocal (study 1) and facial (study 2) displays of distress. We also hypothesized that, as a consequence, there would be a more inconsistent association between arousal and displays in these infants. In study 1, we predicted that lower levels of caregiver responsiveness would be paralleled by less specific infant vocal displays. We predicted that infants from low chaos households would be likely to produce negative affect vocalizations such as cries in clusters, which occur at times when their physiological arousal is elevated. In contrast, we predicted that infants from high chaos households would be more likely to produce intense negative affect vocalizations sporadically, irrespective of their underlying physiological arousal. In study 2, we hypothesized that there would be a similar disconnection between physiological arousal and facial displays in infants from chaotic households during the adapted still face episode.

## METHODS

Confirmatory/exploratory statement: the analyses described below are relatively exploratory in nature. The majority of the analyses described below use methods that have not, to our knowledge, been conducted before.

### Experimental participant details

The project was approved by the Research Ethics Committee at the University of East London. Participants were recruited from the South‐East regions of the UK. In total, 91 infant–caregiver dyads were recruited to participate in the study, of whom usable autonomic data were recorded from 78. A further 4 participants failed to return the chaos questionnaire. Further details, including exclusion criteria and detailed demographic details on the sample, are located in Supporting Information (Section S1.1; Table S1). Of note, we excluded families in which the primary day‐time care was performed by a caregiver who self‐identified as male, because the numbers were insufficient to provide an adequately gender‐matched sample. All participating caregivers, therefore, by caregivers who self‐identified as female. Participants received £30 in Love2Shop gift vouchers as a token of gratitude for participation, split over two visits. Data collection took place between 2018 and 2021.

### Chaos scale

The “chaos” questionnaire (Matheny et al., 1995) asks caregivers to self‐rate on a series of statements such as “it's a real zoo in our home” and “the atmosphere in our home is always calm”. Figure S1 (Section S1.2) shows a histogram of the results obtained from the questionnaire. For the time series analyses reported in parts 2b,c, 3, and 4a–c, where it is not possible to look at group differences based on a continuous group variable, data have been split using a median split, with quartile splits also reported in Supporting Information. The median was 33; Table S1 provides a break‐down of the demographic data split by high/low chaos score, and associations between chaos score and other demographic variables are reported in the results.

### Experimental method details—Home data

Participating caregivers were invited to select a day during which they would be spending the entire day with their child but which was otherwise, as far as possible, typical for them and their child. The researcher visited the participants' homes in the morning (c. 7.30–10 a.m.) to fit the equipment, and returned later (c. 4–7 p.m.) to pick it up. The mean (std) recording time per day was 7.3 (1.4) hours.

**FIGURE 1:**
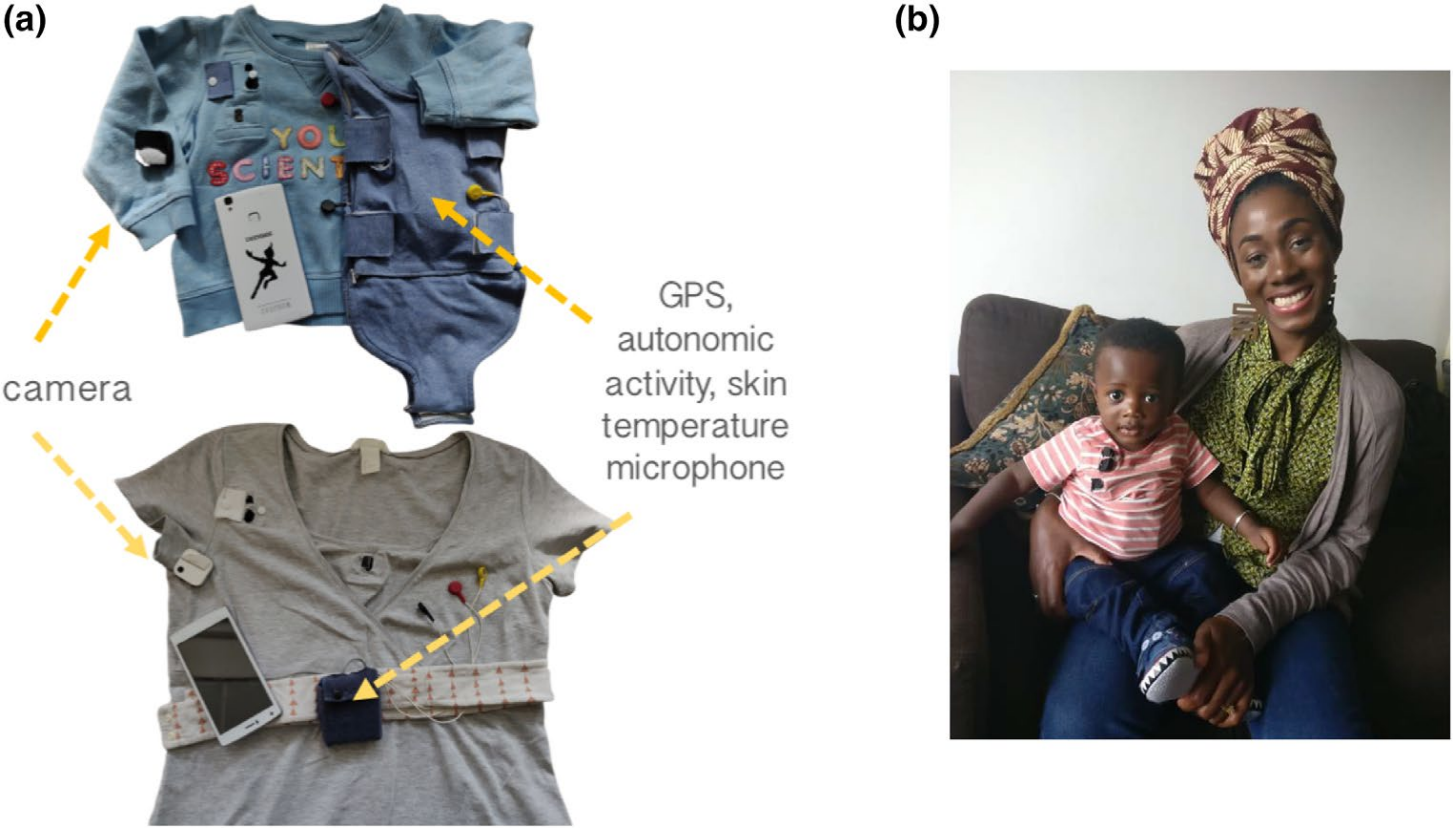
Equipment. (a) Picture showing the equipment used in the study. (b) Picture showing a caregiver and child wearing the equipment. GPS, Global Positioning System.

**FIGURE 2:**
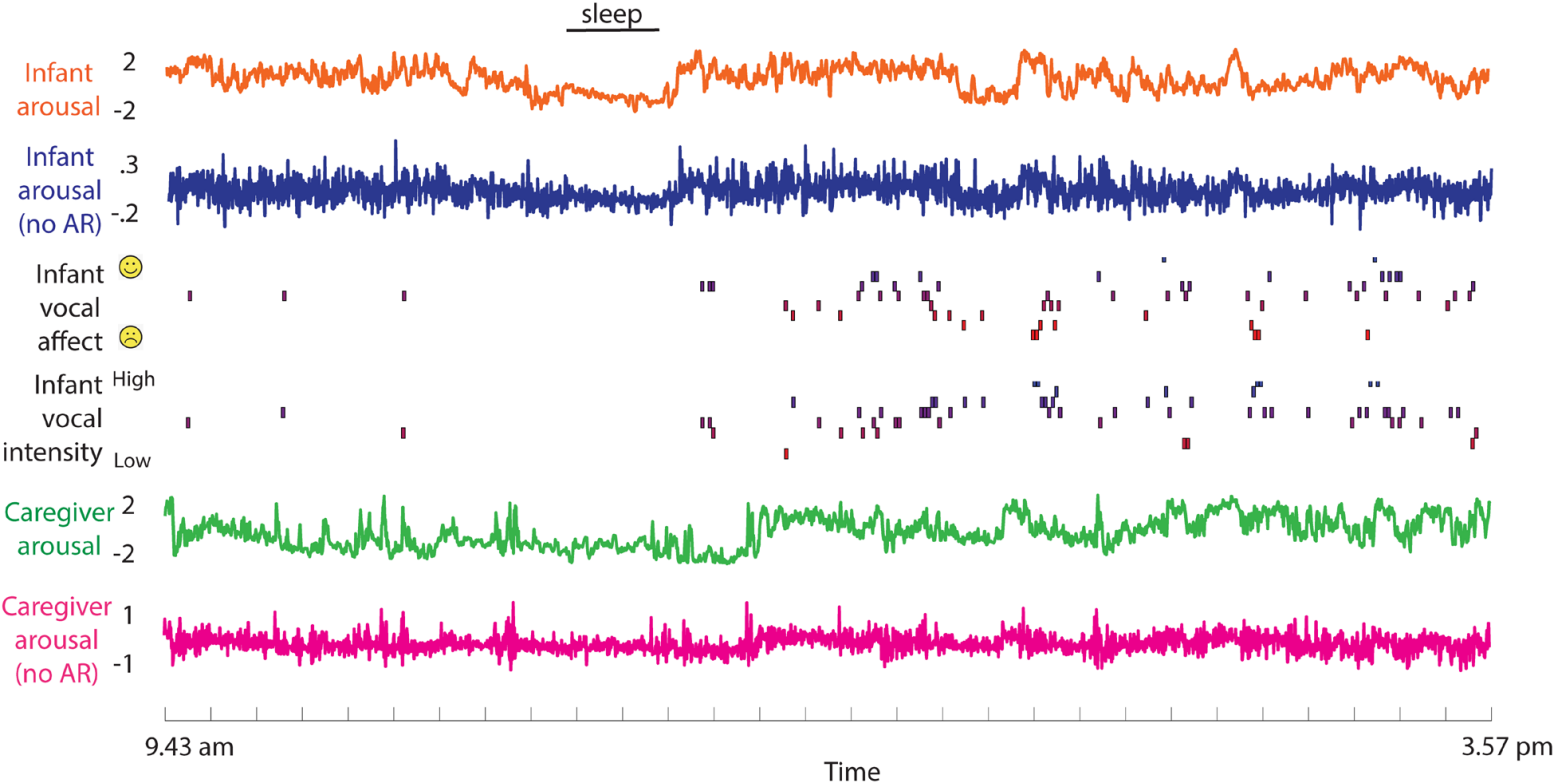
Raw data sample showing, from top to bottom: Infant arousal composite score (see Sections S1.3–S1.6); infant arousal after removal of the autocorrelation (AR; see Section S1.7); infant vocal affect and infant vocal intensity (see Methods section); caregiver arousal; caregiver arousal after removal of the autocorrelation.

The equipment consisted of two wearable layers, for both infant and caregiver (see Figure 1). For the infant, a specially designed baby‐grow was worn next to the skin, which contained a built‐in electrocardiogram (ECG) recording device (recording at 250 Hz), accelerometer (30 Hz), Global Positioning System (1 Hz), and microphone (11.6 kHz) (see Figure 2).

For technical reasons (limited storage capacity), the microphone recorded a 5‐second snapshot of the auditory environment every 60 s. To examine the effect that this had on the temporal patterning of the vocalizations we detected, we conducted a simulation comparison with another dataset (see Section 1.9; Figure S4). This indicated that the temporal patterning of the vocalizations in our data was maintained despite the sparse sampling approach we used.

A T‐shirt, worn on top of the device, contained a pocket to hold the microphone and a miniature video camera (a commercially available Narrative Clip 2 camera). For the caregiver, a specially designed chest strap was also worn next to the skin, containing the same equipment. A cardigan, worn as a top layer, contained the microphone and video camera. The clothes were comfortable when worn and, other than a request to keep the equipment dry, participants were encouraged to behave exactly as they would do on a normal day.

### Quantification and statistical analysis—Home data

#### Autonomic data parsing and calculation of the autonomic composite measure

Further details on the parsing of the heart rate (Section S1.3; Figures S2 and S3), heart rate variability (Section S1.4), and actigraphy (Section S1.5) are given in the Supporting Information. As shown in the Supporting Information (Figure S4), these three variables were highly interdependent, and so we collapsed them into a single composite measure of autonomic arousal (see Section S1.6 for further details). In Section S1.7, we present a description of how the autocorrelation was removed from the arousal data. Because we wished to examine large‐scale arousal changes, all data were down‐sampled to 1‐min epochs for all analyses.

#### Home/awake coding

Our preliminary analyses suggested that infants tended to be strapped in to either a buggy or car seat for much of the time that they were outdoors, which strongly influenced their autonomic data. For this reason, all the analyses presented in the paper only include data segments in which the dyad was at home and the infant was awake. A description of how these segments were identified is given in the Supporting Information (Section S1.8). Following these exclusions, the mean (std) total amount of data available per dyad was 3.7 (1.7) hours, corresponding to 221.5 (102.4) 1‐min epochs per dyad.

#### Vocal affect and intensity coding

Post hoc, trained, blinded coders identified samples in which the infant or caregiver was vocalizing, and coded them on a five‐point scale for vocal affect (negative: fussy and difficult, neutral or positive: happy and engaged), and on a five‐point scale for vocal intensity of the affect expressed, from 1 (least intense) to 5 (most intense). Consistency of rating between coders was achieved through discussions and joint coding sessions based on an *ersatz* dataset, before the actual dataset were coded. To assess inter‐rater reliability, 24% of the sample was double coded. Inter‐rater reliability was assessed jointly for affect and intensity. Cohen's *κ* was 0.60, which is considered acceptable (McHugh, 2012), although it was lower than the reliability observed in some previous studies that conducted similar coding (e.g., 0.71 and 0.62 for the two coding schemes analyzed in Matias et al., 1989). Importantly, however, all coders were blind to study design, participant details and hypothesized study outcome, and the presence of random inaccuracy ought therefore only to have created false negative, not false positive results. In total, a mean (std) of 159 (36) vocalizations per infant was analyzed.

#### Permutation‐based temporal clustering analyses

To estimate the significance of the time‐series relationships in the results, a permutation‐based temporal clustering approach was used. This method examines temporally contiguous patterns of change in instances where the center‐point of the expected response window is unknown, or unimportant (Maris & Oostenveld, 2007). In each case, the test statistic (always specified in the text) was calculated independently for each 1‐min epoch. Series of significant effects across contiguous time windows were identified using an alpha level of .05. 1000 random datasets were then generated with the same dimensions as the original input data. To ensure that the same level of autocorrelation was present in the simulated data as in the original datasets, multivariate autoregressive models were fitted to each sample included in the original dataset using the Matlab function ARfit.m (Neumaier & Schneider, 2001), and the matching AR parameters were used to generate each of the random datasets using the Matlab function ARsim.m (Neumaier & Schneider, 2001). Then, the same sequence of analyses was repeated, and the longest series of significant effects across contiguous time windows was identified. The results obtained from the random datasets were used to generate a histogram, and the likelihood of observed results have been obtained by chance was calculated by comparing the observed values with the randomly generated values using a standard bootstrapping procedure. Thus, a p value of <.01 indicates that an equivalent pattern of temporally contiguous group differences was observed in 10 or fewer of the 1000 simulated datasets created (see e.g. Oakes et al., 2013; Wass et al., 2019 for similar approaches).

#### Control analysis

Participant by participant, for each vocalization that was observed, a random “non‐vocalization” moment was selected as a moment during the day when the dyad was at home and the infant was awake but no vocalization occurred. The same moving window analysis described above was then repeated to examine change relative to this “non‐vocalization event”. The same procedure was repeated 1000 times and the results averaged. Real and observed data were compared using the permutation‐based temporal clustering analyses described above.

### Experimental method details—Lab data

The task was an adapted version of the still face protocol (Weinberg & Tronick, 1996). Caregiver and child were seated across a 80 cm‐wide table, and instructed to play naturally with four toys positioned on the table. After 4 min, on an instruction from the experimenter, the caregiver was instructed not to respond to the infant and to hold a neutral face for 2 min. On a further instruction from the experimenter, the play resumed for a further 2 min. If the infant became distressed during the still face period, as judged using the standard guidelines (Weinberg & Tronick, 1996), the experiment was curtailed. Of note, although the age of the children in the present study is older than the age range typically used for the still face procedure, the task did overall produce increases in arousal in our cohort (as shown below)—meriting its use in this study.

### Quantification and statistical analysis—Lab data

#### Facial and vocal affect coding

Facial and vocal affect was coded in 5‐s bins using a 5‐point scale, where −2 is extreme negative affect, 0 is neutral, and +2 is extreme positive affect. To ascertain inter‐rater reliability, 20% of the sample was double coded, and Cohen's *κ* was calculated. Inter‐rater reliability was found to be 0.66, which is considered acceptable.

#### Autonomic data parsing

The parsing of the ECG data was conducted using the same procedures as used for the home data. Because of technical problems with equipment during the recording of heart rate data from the still face protocol, data from this task are only available from a *N* = 17 subset of the sample. Further details of the parsing of the ECG data are given in the Supporting Information (Section S1.3; Figures S2 and S3).

## RESULTS

This section is structured as follows. First (part 1), we include descriptive statistics, showing how scores on the chaos scale correlated with household noise, demographics, caregiver mental health, tonic arousal, and average vocalization rate affect and intensity.

Second (part 2), we examine caregiver responsivity, by recording how caregivers' arousal changes around infant vocalizations in home settings. In part 2a, we examine how caregivers' arousal changes around infant vocalizations, and how these relationships differ contingent on household chaos. In part 2b, we examine, more specifically, how the infant's arousal level at the time of their vocalization relates to the caregiver's response. In part 2c, we examine how caregivers' vocal behavior changes around infant vocalizations, and how these relationships differ contingent on household chaos.

Third (part 3), we examine infants' vocal communicative behaviors in home settings, and how these relationships differ contingent on household chaos. In part 3a, we examine how infants' arousal changes around vocalizations, and how these relationships differ contingent on household chaos; in part 3b, we examine how the likelihood of an infant producing intense negative affect vocalizations at a given level of arousal differs contingent on household chaos; in part 3c, we examine how likely negative affect vocalizations are to occur in clusters, and how this differs contingent on household chaos.

Fourth (part 4), we examine whether similar patterns as observed in the home are also present in lab settings. We examine infant behavioral and physiological changes during an adapted still face protocol, and how these relationships differ contingent on household chaos.

### Part 1—Descriptives

Before conducting our primary analyses (described from Part 2 onward), we first calculated descriptive statistics to report on: (i) face validity of the chaos scale—association with microphone noise and people in the household; (ii) association of chaos with demographics and caregiver mental health; (iii) association of chaos with tonic arousal; and (iv) association of chaos with vocalization rate, affect and intensity. In addition, in the Supporting Information we also examine the distribution of our chaos scores (Figure S1) and how our high/low chaos groups differ contingent on demographics (Table S1).

#### Face validity of chaos scale—Association with microphone noise levels and people in the household

First, we examined the face validity of the scale by examining the relationship between household noise levels (taken from the average dB levels on the microphones worn by infants) and caregiver household chaos ratings. Because not all variables were normally distributed, more conservative non‐parametric Spearman correlations are reported throughout. A significant association was identified between chaos and microphone levels while the infant was sleeping (*ρ* = .28, *p* = .016), and a marginally non‐significant association was identified between chaos and ambient waking microphone levels (*ρ* = .22, *p* = .069). An association was also observed between chaos and the number of people living in the household (*ρ* = .41, *p* < .001).

#### Demographics and caregiver mental health

We examined the relationship of the chaos scale to the demographic variables recorded: maternal education and household income. In our sample, no significant relationships were identified (all *p*s > .55). In addition, we examined the relationship of the chaos scale to caregiver mental health, as assessed using the Generalized Anxiety Disorder 7‐item screen (Spitzer et al., 2006) and the Patient Health Questionnaire 9‐item screen for depression (Kroenke et al., 2001). A positive association was observed between chaos and caregiver depression (*ρ* = .23, *p* = .026) but not anxiety (*p* = .26).

#### Association with tonic arousal

First, we examined the association between tonic arousal, defined from average heart rate, heart rate variability and movement levels in infant and caregiver. Two categories were examined: segments while the dyad was at home and the infant was awake; and those where the infant was asleep. No significant associations were found between any of these variables and household chaos (all *p*s < .34).

#### Association with vocalization rate, affect and intensity

No association was observed between chaos and average infant vocal intensity (*ρ* = −.07, *p* = .60); a marginally non‐significant positive association was observed between chaos and infant vocal affect (*ρ* = .24, *p* = .084) (higher chaos associated with more positive affect). No association was observed between chaos and the vocalization rate of infants or adults (*ρ* = −.04/0.12, both *p*s > .3), but a marginally non‐significant negative association was noted between chaos and the proportion of adult vocalizations that were infant‐directed (*ρ* = −.22, *p* = .098) (higher chaos associated with a lower proportion of infant‐directed vocalizations).

### Part 2—Home recordings: Household chaos, caregiver responsivity, and infant vocal displays

In this section, we examine caregiver responsivity, by recording how caregivers' arousal changes around infant vocalizations in home settings. First (part 2a), we examine how caregivers' arousal changes around infant vocalizations, and how these relationships differ contingent on household chaos. Second (part 2b), we examine, more specifically, how the infant's arousal level at the time of their vocalization relates to the caregiver's response. Third (part 2c), we examine how caregivers' vocal behavior changes around infant vocalizations, and how these relationships differ contingent on household chaos.

#### Caregiver arousal changes before and after infant vocalizations

**FIGURE 3:**
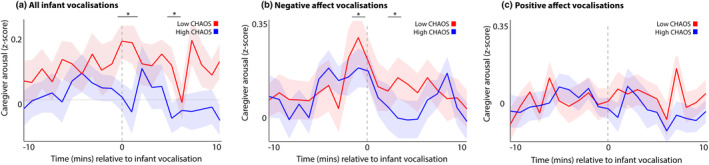
Line graphs showing average caregiver arousal around different types of infant vocalizations, split by median split into children from low and high chaos households. (a) All vocalizations; (b) negative affect vocalizations; (c) positive affect vocalizations. Shaded areas show standard error. *Sections identified as showing significant group differences by the permutation‐based cluster analysis *p* < .05. Chaos, Confusion Hubbub And Order Scale.

In this section, we examine how caregivers' arousal changes around infant vocalizations, and how these relationships differ contingent on household chaos. First, we examined how caregiver arousal patterns change during the period from 10 min before to 10 min after each infant vocalization. (The choice of a length for the time window is arbitrary, and our analyses are designed so that the length of the window can in no way affect the significance of the results.) To analyze the significance of group differences, a permutation‐based clustering analysis was conducted to correct for multiple comparisons, as described in the Methods section. Figure 3 shows the data split using a median split by household chaos. Figure S6 shows the same analysis, but subdivided using a quartile split by household chaos, and Figure S7 shows the same analysis, but divided by caregiver depression, to assess whether group differences were contingent on the association noted above between household chaos and caregiver depression.

For all infant vocalizations (Figure 3a), permutation‐based clustering analyses revealed a significant (*p* = .023) group difference from 0 to +2 min and from +5 to +6 min following the vocalization (Figure 3a), such that caregivers in the high chaos group showed smaller arousal changes around infant vocalizations. For negative affect vocalizations, a significant (*p* = .045) difference was observed from −1 to 0 and from +1 to +2 min relative to infant vocalizations (Figure 3b). For positive affect vocalizations (Figure 3c), no group differences were observed. The results presented in the Supporting Information (Figure S6) show the same analysis repeated using a quartile split by household chaos. These analyses show that the relationship is linear across the sample, and driven by greater caregiver arousal reactivity to infant vocalizations in the bottom quartile (lowest household chaos) subgroup. The results presented in the Supporting Information (Figure S7) show that this relationship is not contingent on caregiver depression.

No significant group differences were observed when we examined caregiver arousal changes to infant vocalizations subdivided by vocal intensity, subdivided by household chaos (Figure S8a,b). However, the same analysis broken down using a quartile split by household chaos (Figure S8c) shows that, again, greater caregiver arousal reactivity to infant vocalizations is observed in the bottom quartile (lowest household chaos) subgroup.

Overall these results suggest that caregivers in the low chaos group show larger changes in autonomic arousal around infant vocalizations, and that this are driven by greater caregiver responsivity to negative affect infant vocalizations.

#### Caregiver arousal changes before and after infant vocalizations—Subdivided by infant physiological arousal

**FIGURE 4:**
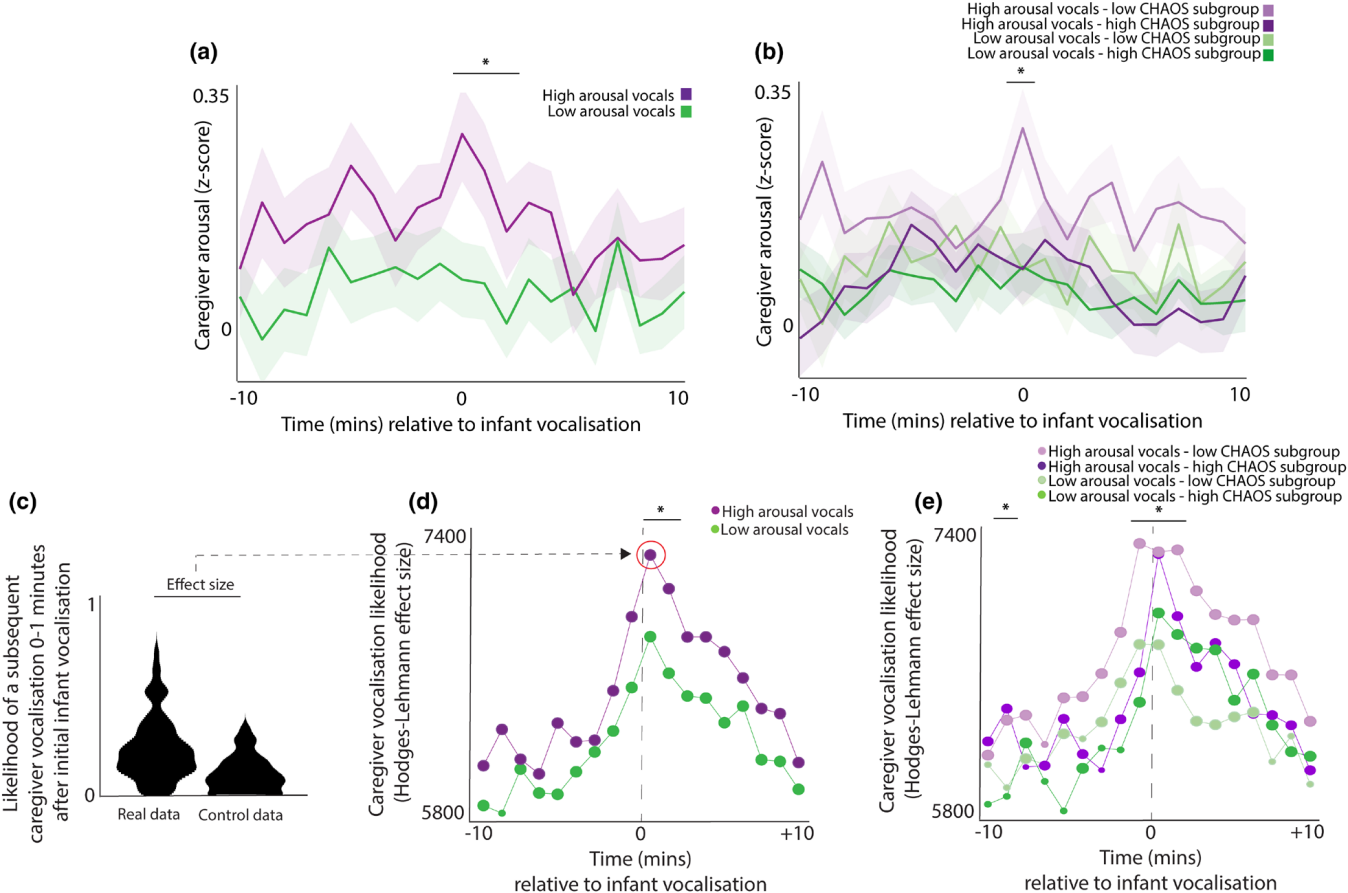
(a) Line graph showing average caregiver arousal around infant vocalizations, split by median split by the infant's arousal at the time of the vocalization. (b) Same figure as (a), but further subdivided by household chaos, to create four categories. For (a, b), shaded areas show standard error. *Sections identified as showing significant group differences by the permutation‐based cluster analysis *p* < .05. (c) Sample violin plot showing the analysis for one time interval that was then repeated iteratively across multiple time intervals in (c). The plot shows the likelihood of a subsequent caregiver vocalization in the time window 0–1 min following an infant vocalization, comparing real with control data. (d) Same analysis repeated across multiple time windows, subdivided between high and low arousal vocalizations. (e) Same analysis repeated across multiple time windows, further subdivided by household chaos. For (c–e), the size of the dot indicates the significance of the Mann–Whitney *U* test comparing the observed and control data. Large dot—*p* > .05; small dot—*p* < .05. *Sections identified as showing a significant group differences by the permutation‐based cluster analysis, *p* < .05. Chaos, Confusion Hubbub And Order Scale.

In this section, we examine, more specifically, how the infant's arousal level at the time of their vocalization relates to the caregiver's response. To do this, we repeated the same analyses as shown in Figure 3, but instead of subdividing vocalizations by household chaos and vocal affect, we subdivided all vocalizations according to the infant's physiological arousal at the time of the vocalization. Separately for each infant we performed a median split to differentiate between high and low arousal vocalizations (relative to the average arousal level for that child). The results are shown in Figure 4a. The same permutation‐based temporal clustering analysis was performed as described above. This suggested that high arousal vocalizations were accompanied by significantly (*p* < .001) greater changes in caregiver arousal during the time period from 0 to +3mins after the vocalization. We then further subdivided these vocalizations by household chaos, to create four categories—high arousal vocalizations from the low chaos group, high arousal vocalizations from the high chaos group, low arousal vocalizations from the low chaos group, and low arousal vocalizations from the high chaos group. We found that only the low chaos group caregivers appeared to respond differentially to high arousal vocalizations (Figure 4b).

Overall, these results suggest that caregivers' arousal changes are greater following high arousal infant vocalizations compared with low arousal vocalizations, and that only the low chaos group caregivers appeared to respond differentially to high arousal vocalizations.

#### Caregivers' vocal responsivity

In this section, we examine how caregivers' vocal behavior changes around infant vocalizations, and how these relationships differ contingent on household chaos. To do this, we used the following procedure: for each vocalization, we calculated the average likelihood of another infant vocalization during a given 1‐min time window (e.g., 1–2 min following an initial infant vocalization—see Figure 4c). To estimate whether the observed likelihood differed from chance we performed a control analysis in which we inserted random “non‐vocalization” events into the data and repeated the analysis relative to these “non‐vocalizations”, and compared the “real” and “control” datasets using a nonparametric Mann–Whitney *U* test (see Figure 4c). We then repeated this analysis across multiple time windows from 10 min before the vocalization to 10 min after (Figure 4d). Again, the choice of time window is arbitrary and our analyses are designed so that the length of the time window cannot have influenced our results.

Figure 4d shows caregiver vocalization likelihood subdivided between high and low arousal infant vocalizations. The size of the dots in Figure 4d indicates the significance of the statistical analyses comparing the observed vocalization rate with chance. The results suggested that, in all bins examined (apart from −9 mins for low arousal vocals) the observed vocalization rates were greater than the baseline levels. In addition, the observed vocalization rates were directly compared between the two conditions. Permutation‐based temporal clustering analyses suggested that caregivers are significantly more likely to vocalize during the time period 0 to +2 mins following high arousal vocalizations.

Finally, we examined how caregiver's vocal behavior around infant vocalizations differs contingent on household chaos. To do this, we calculated the same plot as shown in 4d, but further subdivided by household chaos (Figure 4e). Our analyses suggested that caregivers from the low chaos group are significantly more likely to vocalize during the time period −2 to +2 mins around high arousal vocalizations. Overall, these results suggest that caregivers are more likely to vocalize in the time period following high arousal infant vocalizations, with the highest response rate observed from caregivers in the low chaos subgroup in response to high arousal vocalizations.

### Part 3—Home recordings: Household chaos and infant → caregiver communication

In this section, we examine infants' vocal communicative behaviors in home settings, and how these relationships differ contingent on household chaos. First, in part 3a, we examine how infants' arousal changes around vocalizations, and how these relationships differ contingent on household chaos. Second, in part 3b, we examine how the likelihood of an infant producing intense negative affect vocalizations at a given level of arousal differs contingent on household chaos. Third, in part 3c, we examine how likely negative affect vocalizations are to occur in clusters, and how this differs contingent on household chaos.

#### Arousal changes before and after vocalizations

**FIGURE 5:**
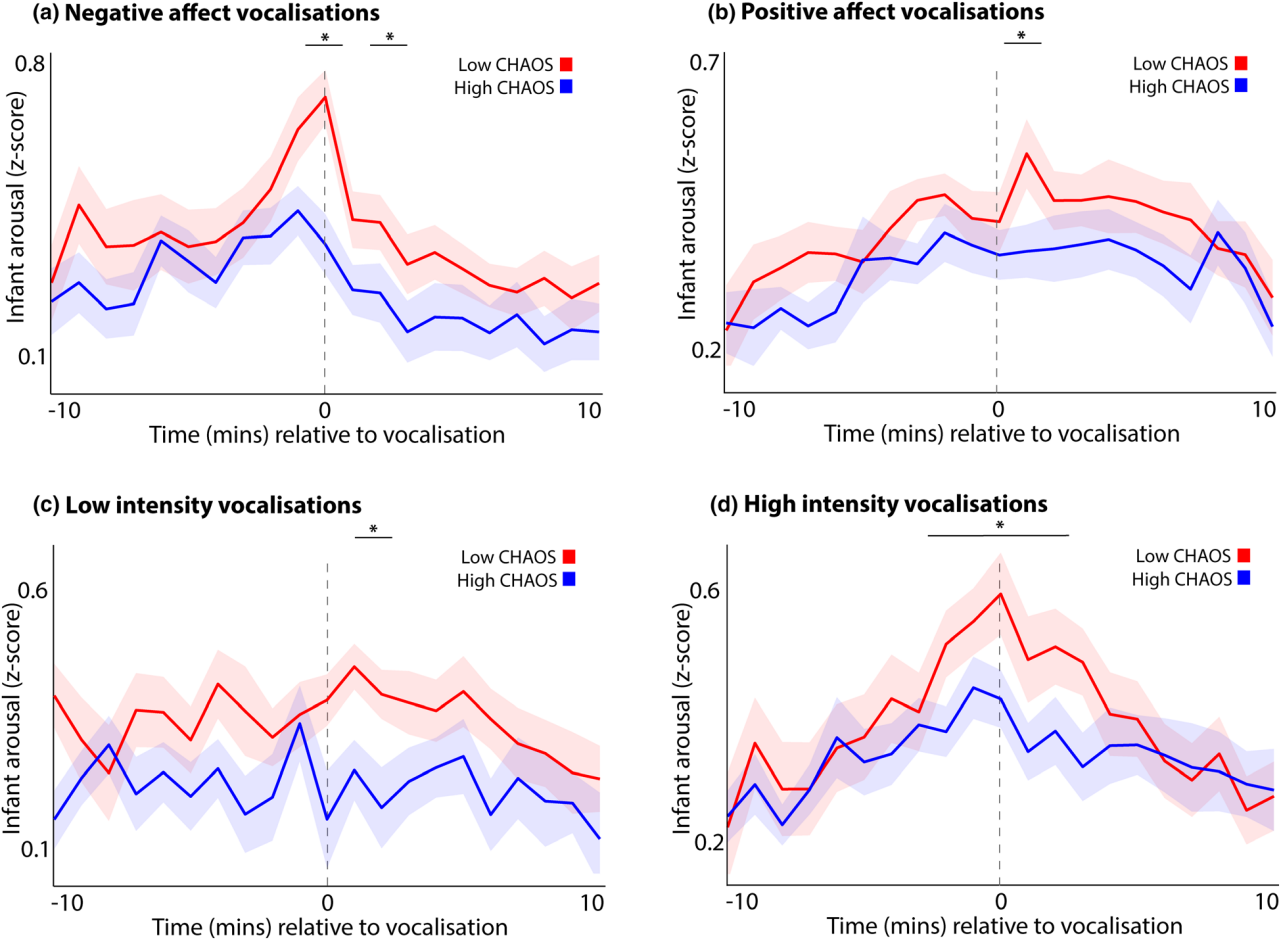
Line graphs showing average infant arousal around different types of vocalizations, split by median split into children from low (blue) and high (red) chaos households. (a) Negative affect vocalizations; (b) positive affect vocalizations; (c) low intensity vocalizations; (d) high intensity vocalizations. Shaded areas show standard error. *Sections identified as showing significant group differences by the permutation‐based cluster analysis *p* < .05. Chaos, Confusion Hubbub And Order Scale.

In this section, we examine how infants' arousal changes around vocalizations, and how these relationships differ contingent on household chaos. First, we calculated the average change in infant arousal around all negative (Figure 5a) and positive affect infant vocalizations, subdivided using a median split into low/high chaos groups. In the Supporting Information, we also include the same analysis based on a quartile split by household chaos, to examine how continuously these group differences were observed in our data (see Figure S9).

For negative affect vocalizations, two significant (*p* = .001) clusters were found: one between −1 to +1 min, and one from +4 to +5 min following the vocalization (Figure 5a), suggesting that the high chaos group showed smaller arousal changes around vocalizations. For positive affect vocalizations, a significant (*p* = .025) difference was observed from +1 to +2 min following the vocalization (Figure 5b). For low intensity vocalizations, a significant (*p* = .018) difference was observed from +2 to +3 min following the vocalization (Figure 5c); for high intensity vocalizations a significant (*p* = .025) difference was observed from −3 to +3 min around vocalizations. In each case, the direction of these effects was the same: the high chaos group showed smaller arousal changes around vocalizations. The results presented in the Supporting Information (Figure S8) show that, when the analysis is repeated using a quartile split by household chaos, the relationship is continuous across the sample.

Overall, these results show that infants in the low chaos group show larger changes in autonomic arousal across all types of vocalizations; differences appear most marked for negative affect, and high intensity vocalizations. This suggests that there is a disconnection between vocal displays and physiological arousal in the high chaos group.

#### Stacked bar charts

**FIGURE 6:**
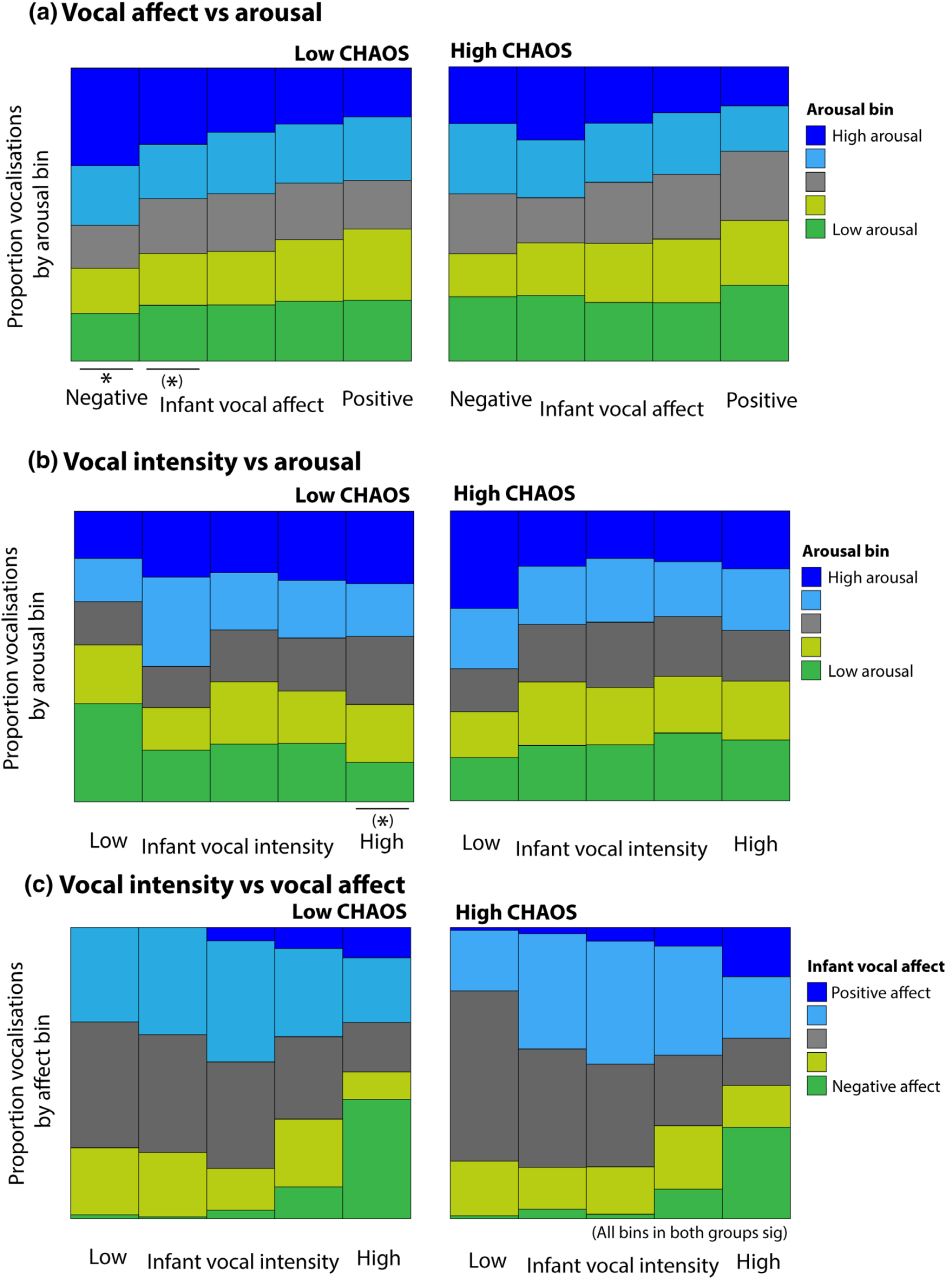
Stacked bar charts showing: (a) the relationship between vocal affect and arousal at the time of vocalization, split between the low (left) and high (right) chaos groups. *Significance of the statistical analyses reported in the main text *p < .05 after correction for multiple comparisons; (*)—*p* < .05 before but not after correction; (b) the relationship between vocal intensity and arousal at the time of vocalization, split between low (left) and high (right) chaos groups; (c) the relationship between vocal intensity and vocal affect, split between low (left) and high (right) chaos households. For (c), statistical analyses showed that all bins in both groups were significant. Chaos, Confusion Hubbub And Order Scale.

In this section, we examine how the likelihood of an infant producing intense negative affect vocalizations at a given level of arousal differs contingent on household chaos. To examine this, we first subdivided our vocalizations by vocal affect and physiological arousal, and plotted two separate stacked bar charts for the low and high chaos groups, determined by a median split (Figure 6a). In the Section S2.4 (Figure S10), we also include the same plots subdivided using a quartile split.

Statistical analyses were conducted by calculating a three‐way ANOVA with the design: percentage vocalizations ~ vocal affect bin × arousal bin × household chaos (treated as a continuous measure). This indicated that the three‐way interaction was not significant *F* = 1.07, *p* = .24, but that the two‐way interactions between household chaos and arousal was significant *F* = 3.07, *p* < .001, such that in the low household chaos group more vocalizations overall were observed at higher arousal. In addition, as a preliminary investigation, we also conducted post hoc analyses in which we calculated separate one‐way ANOVAs with the design: percentage vocalizations ~ arousal bin, separately for each vocal affect bin and each group split by household chaos (see Figure 6a). We corrected for multiple comparisons using the Benjamini–Hochberg procedure (Benjamini & Hochberg, 1995). In the low chaos group, we observed a significant relationship between negative affect vocalizations and arousal *F*(4,134) = 6.7, False Discovery Rate‐corrected *p* = .006, such that extreme negative affect vocalizations are more likely at elevated arousal. This relationship is not present in the high chaos group (*p* = .62). For moderate negative affect vocalizations in the low chaos group, the same relationship was present but did not survive correction for multiple comparisons (*p* = .074). Again, this relationship is absent in the high chaos group (*p* = .62). In the Supporting Information (Figure S10), we present the same analysis based on a quartile (rather than median) split by chaos, showing that this relationship is observed across all quartiles and is most marked in the bottom (lowest chaos) quartile group. Overall, these results suggest that, in the low household chaos group, more vocalizations overall were observed at higher arousal, and that this relationship is specifically driven by differences around negative affect vocalizations.

Figure 6b examines the correspondence between vocal intensity and arousal. We conducted the same 3‐way ANOVA as described above: percentage vocalizations ~ vocal intensity bin × arousal bin × household chaos. This identified a significant 3‐way interaction between household chaos, arousal and vocal intensity *F* = 1.3, *p* = .003, along with the same two‐way interaction between household chaos and arousal described in the first analysis. To understand what drove this three‐way interaction, we also conducted the same post hoc analyses as described above. In the low chaos group, extremely intense vocalizations are more likely at elevated arousal, although this relationship did not survive correction for multiple comparisons (post‐correction *p* = .082). This relationship is absent in the high chaos group (*p* = .92). Although differences also appeared evident for low intensity vocalizations, these were non‐significant (which could be due to the relatively lower frequency of this type of vocalization in our data). Again, in the Supporting Information (Figure S10), we present the same analysis based on a quartile split by chaos, showing that the relationship is continuous across the sample.

Finally, in Figure 6c, we examine the relationship between vocal affect and intensity. This shows that, for both groups, extreme negative and positive affect vocalizations are more common at high vocal intensity, which rules out the possibility that the findings reported above were due to differential coding of vocal affect and intensity in the two groups (e.g., because vocalizations are more difficult to identify in the high chaos group). ANOVAs showed highly significant associations between affect and intensity across all bins examined (all *p*s < .001); no differences contingent on chaos were observed.

Overall, these results suggest that, in the low household chaos group, more vocalizations overall were observed at higher arousal, and that this relationship seems specifically driven by differences around negative affect vocalizations.

#### Temporal clustering of infants' vocalizations

Finally, we examined how likely infant vocalizations are to occur in clusters, and how this differs contingent on household chaos. To do this, we used the same approach as described in section 2c above; but, instead of examining the likelihood of caregivers vocalizing around infant vocalizations, we instead examined the likelihood of infants vocalizing again during the time period before and after their own vocalizations.

**FIGURE 7:**
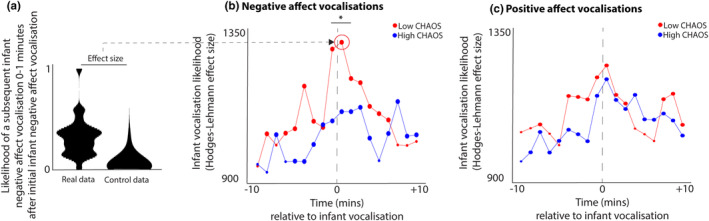
(a) sample violin plot showing the analysis for one time interval that was then repeated iteratively across multiple time intervals in (b). The plot shows the likelihood of a subsequent infant negative affect vocalization in the time window 0–1 min following an infant negative affect vocalization, comparing real with control data. (b) Same analysis repeated across multiple time windows, subdivided between low (red) and high (blue) chaos households. For each time window, the size of the dot indicates the significance of the Mann–Whitney *U* test comparing the observed and control data. Large dot—*p* > .05; small dot—*p* < .05. *Sections showing significant group differences according to the permutation‐based cluster analysis *p* < .05. Chaos, Confusion Hubbub And Order Scale.

For each time window, the significance of each Mann–Whitney *U* test comparing the observed vocalization rate with chance is shown in Figure 7b: a large dot indicates a significant difference between the observed and the control data (i.e. that a vocalization was significantly more likely to occur during that time window compared with chance). Our results showed that, in the low chaos group, a significantly greater than chance vocalization likelihood was observed from 9 min before each vocalization to 7 min after (although not all intermediate bins were significant—see Figure 7b). In the high chaos group, greater than chance vocalization rates were observed from 8 mins before to 10 mins after (although again not all intermediate bins were significant).

In addition, we also directly compare the vocalization rates between the high and low chaos groups, using a Mann–Whitney *U* test and a permutation‐based clustering analysis to correct for multiple comparisons. This test identified a significantly higher vocalization rate (*p* < .001) in the low chaos group from −1 to +1 mins following the vocalization, suggesting higher clustering for negative vocalizations in this group.

Figure 7c shows and identical plot of positive affect vocalizations. In the low chaos group, greater than chance vocalization likelihoods were observed between 8 min before each vocalization to 10 min after (although again not all intermediate time windows were significant). In the high chaos group, greater than chance vocalization rates were observed between 9 mins before to 10 mins after. No direct group comparisons were significant, suggesting no differences in clustering for positive vocalizations.

Overall these results suggest that, in the low chaos group, negative affect infant vocalizations are more likely to be accompanied by other infant vocalizations during the period immediately around the event. No significant group differences were observed for positive affect vocalizations.

### Part 4—Lab recordings: Household chaos, infant behavior and physiology during an adapted still face paradigm

**FIGURE 8:**
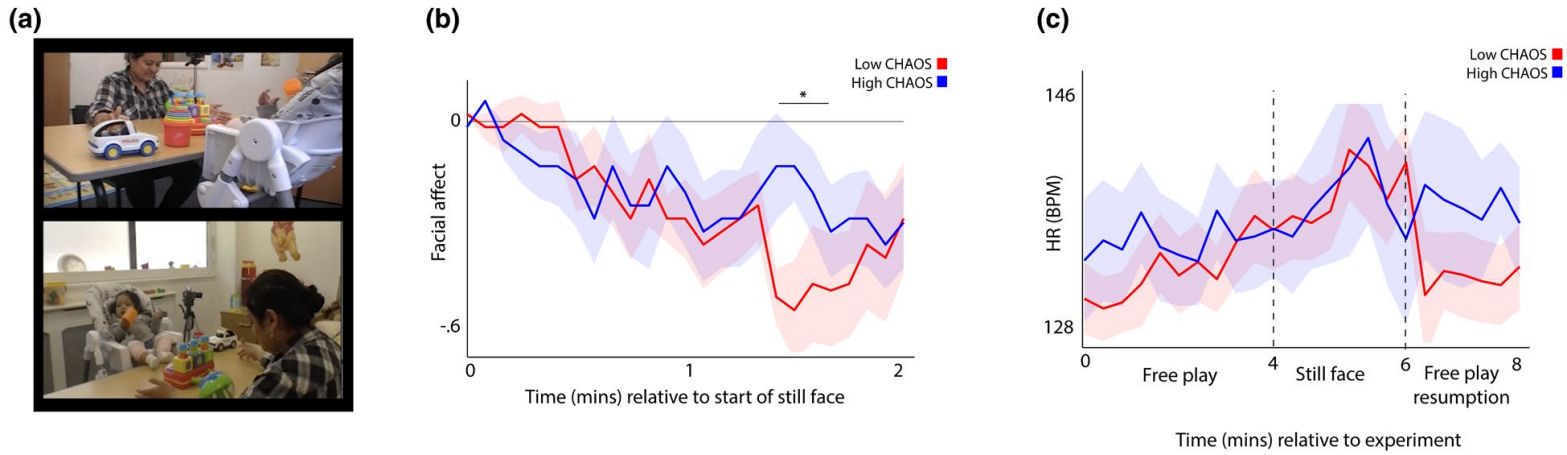
(a) screen grabs illustrating the experimental set‐up; (b) line graph showing change in facial affect during the adapted still face, subdivided by high and low household chaos; (c) line graph showing change in heart rate during all three phases of the experiment, subdivided by high and low household chaos. Shaded areas show standard error. *Sections identified as showing significant group differences by the permutation‐based cluster analysis *p* < .05. Chaos, Confusion Hubbub And Order Scale.

In this section, we examine infant behavioral and physiological changes during an adapted still face protocol, and how these relationships differ contingent on household chaos. Figure 8a shows the experimental set‐up that we used for the adapted still face procedure. First, we examined the change in infant facial affect during the procedure, subdivided into high and low chaos groups (Figure 8b). The same permutation‐based temporal clustering analysis as described above was applied to test for group differences while correcting for multiple comparisons. This identified a significant (*p* = .034) difference in facial affect in the time period 1:20 to 1:40 during the still face, such that the high chaos group showed less negative affect. By contrast, when we examined physiological changes, we found no significant difference between groups (although the *N* for this analysis is lower, as described in the Methods section). However, we did identify a trend‐level correlation between chaos and heart rate recovery following the still face (*ρ* = .47, *p* = .056), such that higher household chaos associated with slower recovery following the still face. Overall, these results suggest that infants from high chaos households display a tendency toward less negative facial affect during the still face and for slower physiological recovery afterward.

## DISCUSSION

Using day‐long home recordings obtained using miniature wearable autonomic monitors and microphones, we examined caregiver‐infant physiological arousal and vocal affective displays in high‐ and low‐chaos households. We examined within‐individual relationships (how infant autonomic arousal relates to infant vocal affect) and cross‐dyad relationships (how caregiver arousal relates to infant vocalizations). We also examined differences in facial displays and autonomic arousal in infants from the different households during the adapted still face paradigm.

First, our results suggested that caregivers from high chaos households show smaller arousal changes around infant vocalizations (Figure 3a), in particular around negative affect vocalizations (Figure 3b). When we examined how caregiver responsivity varies as a function of infant arousal at the time of the vocalization (Figure 4) we found that all caregivers are less responsive to low arousal vocalizations (Figure 4a,d). However, when we subdivided this by household chaos we found that, whereas low chaos caregivers did differentiate between high and low arousal vocalizations, high chaos caregivers did not (Figure 4b,e). In other words, infant arousal at the time of the vocalization influenced the likelihood of a caregiver response in low chaos households, but the same relationship was not observed in high chaos households.

Second, when we examined infants' vocal communicative behaviors in home settings, we found across three analyses that infants from low chaos households are more likely to produce negative affect vocalizations such as cries in clusters, which occurred at elevated arousal (Figures 5, 6, 7); however, this relationship is absent in the high chaos group. The findings from the low‐chaos group are consistent with previous research in general populations (i.e. not subdivided by household chaos level) (Kreibig, 2010; Wass et al., 2022) which show that negative vocalizations occur most often at high arousal states; they are consistent with the idea that initially, infant vocalizations are not functionally flexible, but largely determined by levels of arousal (Ghazanfar & Zhang, 2016; Wass et al., 2022; Zeskind, 2013). By contrast, our results suggest that infants from more chaotic households are not using high intensity vocalizations to communicate their high arousal states. This reveals a disconnect between their levels of physiological arousal—which are likely to reflect what they are experiencing—and what they are actually expressing. This means that, in the first year of life, vocal flexibility with respect to physiological arousal may already be modulated by contextual factors and caregiver responsivity.

One possibility is that the differences in child behavior we observed may be attributable to other factors, such as the proximity between child and caregiver, and/or the presence of other children and adults at home. Regrettably, we did not measure child–caregiver proximity and the presence of other people at home during our recordings in this study. However, the fact that similar differences were also observed in study 2, which was conducted in controlled lab settings, partially counts against this possibility.

One alternative possibility is that the differences in child communicative behavior we observed may be more attributable to other factors, such as child temperament. Rather than it being the case that the child's home environment influences the caregiver's behaviors and, through that, the child's communicative behaviors toward their caregiver, it is possible that the differences in communicative behaviors we observed may be attributable to other factors, such as child temperament, which happen to co‐vary with household chaos. Importantly, however, we found no evidence that infants in the high‐chaos group are experiencing different autonomic arousal, or vocalizing differently overall. We also found no evidence that arousal was higher overall in infants from the high chaos group, or that more negative affect overall was observed in the high chaos group; and we observed only a trend toward slower physiological recovery from still face procedure in the high chaos group. Finally, we observed no relationship between household chaos and infant vocalization rates, and no relationship between household chaos and overall vocalization intensity or vocal affect. At this age, the difference we observed between the two groups mostly concerned signaling: how infants' affective displays actually relate to their underlying physiological states.

Taken together, our findings suggest that in chaotic households, caregiver‐infant communication is atypical. This might be due to higher levels of noise in the sonic environment, to reduced rhythmic patterns in communicative exchanges, to a cramped physical environment leading to differences in the physical proximity between child and caregiver, and/or to differences in the visual access of the caregiver to the child, or most likely a combination of these factors. This, in turn, might impact infants' tendency to signal their states of arousal through specific displays: since their displays are not as tightly connected to specific responses from caregivers, infants in more chaotic households might fail to learn that their displays can have specific effects on caregivers, or alternatively, they may learn more quickly than infants in less chaotic households to disconnect their displays from physiological arousal. This effect may be mutually reinforcing as infants signal less to their caregivers, who consequently have fewer signals to respond to and are thus less likely to reverse this trajectory.

This is relevant to theories of selective reinforcement in communication (Albert et al., 2018; Goldstein & Schwade, 2008; Locke, 2006; Oller & Griebel, 2020; Zhang & Ghazanfar, 2016). Our findings suggest that specific signals may lose the potential functional significance that we assume they had in earlier infancy for children in high chaos households, potentially resulting in negative affect vocalizations such as cries becoming less specific. Consistent with this is our finding that, in the low chaos group, negative affect vocalizations such as cries are more likely to occur at elevated arousal, but this relationship is absent in the high chaos group (Figure 6a). Possibly this indicates that, in contrast with previous studies in general populations which found that negative affect vocalizations were more likely to occur at high arousal states (Tronick, 2007; Wass et al., 2019, 2022), these infants have learned that expressing distress when aroused does not have consequences, in the sense of eliciting arousal co‐regulation.

Interestingly, we also found some evidence for a similar disconnection between affective displays and physiological arousal in a lab‐based adapted still face protocol: children from high chaos households showed less negative facial affect (Figure 8b). This contrasts with previous selective reinforcement interpretations of the still face paradigm in general populations, in which infants are said to increase their vocal and facial displays to gain a previously conditioned response from their caregiver (Ham & Tronick, 2009). For instance, Goldstein and colleagues found that 5‐month‐old infants responded to their caregiver's neutral face with increased behavioral signals (clusters of vocalizations) followed by quietness. The authors interpreted this as a classic extinction burst (Goldstein et al., 2009).

Here, our findings mirror those in the more naturalistic data: infants from high chaos households show similar levels of arousal during the adapted still face; but, in contrast with children from low chaos households, they do not match this with their facial display. There is again a disconnection between their physiological response and their communicative behavior. This could mean that there is no/under regulation by the caregiver of their child's distress during the post‐still face; or, it could indicate that this behavior had perhaps not been previously reinforced; and so facial affect too has possibly lost potential functionality (Conradt & Ablow, 2010; Enlow et al., 2014; Feldman, Singer, et al., 2010; Gunning et al., 2013; Haley & Stansbury, 2003; Ham & Tronick, 2009; Provenzi et al., 2015). Our autonomic data show the physiological correlates of this, indicating that infants from high chaos household showed a trend toward slower recovery of their arousal level (return to baseline) during the post‐still face period. Of note, though, the sample for this last analysis was lower than the other analyses in this paper.

Taken overall, our findings thus reveal a disconnect between the arousal patterns in infants in high chaos households, and their vocal communicative behaviors. This disconnect is not seen in infants from low chaos households. Previous research has highlighted the relationship between caregiver–child arousal levels (arousal coupling) as an important mechanism for co‐regulation across the dyad (Feldman, 2007; Smith et al., 2021; Wass et al., 2022). In part, infant arousal levels are communicated by vocalizations to elicit a caregiver response (Ghazanfar & Zhang, 2016; Oller & Griebel, 2020): infants are able to utilize their vocalizations to regulate their arousal levels (and overall arousal level across the child–caregiver dyad). If communication is in effect the sending and receiving of signals, the fundamental starting point must be that the overall outcome is beneficial to both partners, in the sense of allowing for effective co‐regulation to take place (Planer & Godfrey‐Smith, 2021; Smith & Harper, 2003; Wass et al., 2024). Our data indicate that in highly chaotic households, both communicative and responsive behaviors are atypical: infants are not expressing their levels of arousal, and caregiver arousal levels are under‐responsive to their infants.

Although technical factors meant that we were confined to random sampling during the day rather than continuous recordings, our analyses examine event‐related changes relative to vocalizations, and so the presence of undetected vocalizations only seems likely to weaken the patterns of event‐related change that we have observed. Furthermore, analyses presented in the Supporting Information (Section S1.9) indicate that the temporal patterning of vocalizations was preserved despite the spare sampling approach that we used. Another limitation (and strength) of our approach is that, although we only included data segments recorded while the dyad was at home and the infant was awake, our home‐based recordings nevertheless contained all types of vocalizations across a variety of physical settings.

In future, it would be interesting to explore the relationship between autonomic arousal, vocalizations, caregiver responsivity, and psychopathology. For example, one factor that may mediate our present findings is caregiver depression, which was positively associated with household chaos in our sample (see also Nelson et al., 2009). Previous research has shown that more depressed mothers tend to be under‐responsive to their infants' vocalizations and behaviors (Beebe et al., 2008; Field et al., 1990). Household chaos has previously been found to act as a mediator between caregiver depression and child outcomes (Hur et al., 2015). One further explanation for our findings could be the higher levels of stress experienced by adults in high chaos households (Bodrij et al., 2021), and associated lower levels of sensitivity (Andeweg et al., 2020). Yet, we note that there was no association with anxiety in our sample.

Overall, our present results suggested that, in infants from high chaos households, infant vocalizations may lose the functional significance that they originally held; and that this is paralleled by decreased caregiver responsiveness; meaning that autonomic regulation following a stressor was slower. But other work has shown that, in a general population, moments of high infant arousal were more likely to be accompanied by infant vocalizations and by high intensity and negative affect vocalizations, including cries and that this served as a mechanism for co‐regulation across the dyad (Wass et al., 2022). Understanding how arousal level and vocalizations likelihood relate to caregiver responsivity across typical and atypical development is an important goal for future research. Relatedly, longitudinal studies are needed to examine the association between infant arousal, vocalizations, and caregiver responsivity across development; how, for example, do vocalizations become functionally flexible over time, what is the relationship between functional flexibility and arousal, and how is this affected by caregiver responsiveness and psychopathological symptoms?

In summary, our data suggest that caregiver responsiveness and infants' communication of their distress may be associated with one another, and that this influence may lead to adverse outcomes in high chaos households. In these households, infant vocalizations (in particular negative vocalizations and high intensity vocalizations) are not more likely to occur at times when infant arousal is elevated, and they do not elicit responsiveness in the caregiver. Our findings also suggest that vocalizations may drive of co‐regulation: when sender–receiver communicative behaviors are finely attuned, which seems to be favored by a less chaotic environment, vocalizations elicit caregiver responsiveness, aiding recovery in the infant.

## CONFLICT OF INTEREST STATEMENT

The authors declare that they have no conflicts of interest.

## Supporting information


Data S1.

## Data Availability

Due to the personally identifiable data of the data contained in this manuscript (microphone recordings of infants), access to the data is only available through direct request to the first author. All of the analytic code necessary to reproduce the analyses presented in this paper is publicly accessible, on request from the corresponding author. The analyses presented here were not preregistered.
