# Supplementary material for: Needing to shout to be heard? Caregiver under‐responsivity and disconnection between vocal signaling and autonomic arousal in infants from chaotic households
Source: Child Dev. 2024 Nov 8;96(2):527–45. doi: 10.1111/cdev.14183 (PMC11868693; doi:10.1111/cdev.14183)
Supplement: Supplementary file 1 — Data S1. [file CDEV-96-527-s001.docx]

Supplementary Materials for:

**Needing to shout to be heard? Affective dysregulation, parental under-responsivity, and a disconnection between vocal signalling and autonomic arousal in infants from chaotic households**

Table of Contents

[1 Supplementary Methods 2](#_Toc156835495)

[1.1 Experimental participant details 2](#_Toc156835496)

[1.3 Heart rate data 3](#_Toc156835497)

[1.4 Heart-Rate Variability (HRV) 6](#_Toc156835498)

[1.5 Actigraphy 6](#_Toc156835499)

[1.6 Arousal composite 7](#_Toc156835500)

[1.7 Removal of autocorrelation from arousal data 8](#_Toc156835501)

[1.8 Home/Awake coding 8](#_Toc156835502)

[1.8.1 Home/not home 8](#_Toc156835503)

[1.8.2 Sleeping/waking 8](#_Toc156835504)

[1.9 Supplementary analysis – simulation to examine the effects of sparse sampling on the data. 9](#_Toc156835505)

[2 Supplementary Results 10](#_Toc156835506)

[2.1 Part 2 – repeated with quartile split by household chaos 10](#_Toc156835507)

[2.2 Part 2 – repeated with median split by caregiver depression 10](#_Toc156835508)

[2.2 Part 2 - Caregiver arousal changes around low- and high-intensity infant vocalisations 10](#_Toc156835509)

[2.3 Part 3a – repeated with quartile split by household chaos 11](#_Toc156835510)

[2.4 Part 3b – repeated with quartile split by household chaos 12](#_Toc156835511)

# Supplementary Methods

## Experimental participant details

This sample size was selected prior to the commencement of the study based on power calculations presented, and approved by peer review, in the funding application that supported this work (XXXX). Exclusion criteria included: complex medical conditions, skin allergies, heart conditions, parents below 18 years of age, and parents receiving care from a mental health organisation or professional. Full demographic details of the participants are given below.

|  | | Whole sample | Low CHAOS | High CHAOS |
| --- | --- | --- | --- | --- |
| N | | 74 | 37 | 37 |
| Infant age (days) – mean | | 354.5 | 353.7 | 355.3 |
| - *SE* |  | 4.6 | 6.8 | 7.8 |
| Gender (% male) | | 39.3 | 37.8 | 43.2 |
|  |  |  |  |  |
| Infant Ethnicity (%) | White British | 50.0 | 48.6 | 51.4 |
|  | Other white | 10.5 | 8.1 | 13.5 |
|  | Afro-Caribbean | 9.2 | 13.5 | 5.4 |
|  | Asian, Indian & Pakistani | 10.5 | 13.5 | 8.1 |
|  | Mixed - White/Afro-Carib | 2.6 | 2.7 | 2.7 |
|  | Mixed - White/Asian | 7.9 | 8.1 | 8.1 |
|  | Other mixed | 9.2 | 5.4 | 10.8 |
|  |  |  |  |  |
| Household Income (%) | Under £16k | 30.3 | 37.8 | 24.3 |
|  | £16-£25k | 27.6 | 27.0 | 27.0 |
|  | £26-£35k | 11.8 | 10.8 | 13.5 |
|  | £36-£50k | 11.8 | 13.5 | 10.8 |
|  | £51-£80k | 10.5 | 2.7 | 16.2 |
|  | >£80k | 7.9 | 8.1 | 8.1 |
|  |  |  |  |  |
| Maternal education (%) | Postgraduate | 32.9 | 43.2 | 21.6 |
|  | Undergraduate | 50.0 | 40.5 | 59.5 |
|  | FE qualification | 2.6 | 5.4 | 0 |
|  | A-level | 3.9 | 2.7 | 5.4 |
|  | GCSE | 5.3 | 8.1 | 2.7 |
|  | No formal qualifications | 2.6 | 0 | 5.4 |
|  | Other | 1.3 | 0 | 2.7 |

*Table S1: Demographic details for: a) the whole sample; b) and c) – data subdivided into low/high CHAOS groups using a median split, as described in the main text.*

1.2 CHAOS questionnaire results


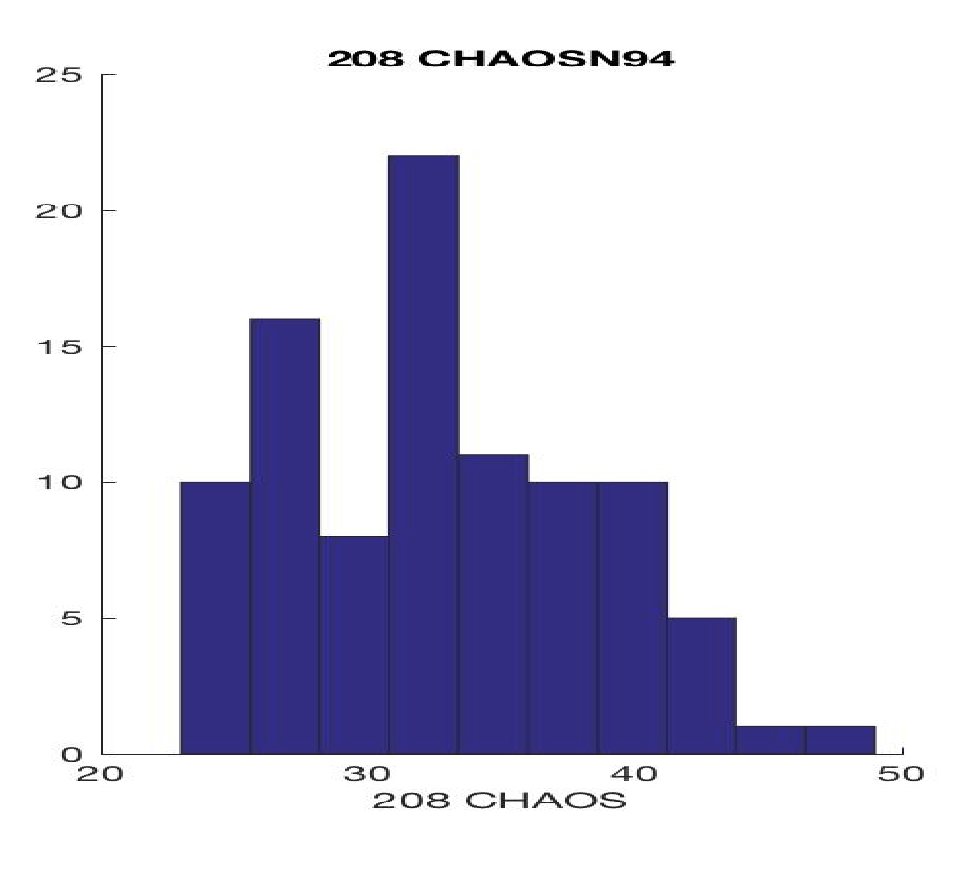


*Figure S1: Histogram of CHAOS questionnaire results*

## Heart rate data

ECG was recorded at 250Hz. To ensure good quality recordings, the ECG device was attached using standard Ag-Cl electrodes, placed in a modified lead II position. Due to technical problems with the ECG recording leads (N=9) and to problems with attaching the ECG recording electrodes securely (N=2), the ECG data were unavailable for 11 of the 93 participants originally tested.

To ensure the accuracy of these recording devices, they were cross-validated by recording heart rate and heart rate variability using both the new devices at home and established recording devices (a Biopac MP150 amp recording at 2000Hz) in lab settings. High reliability was observed both for heart rate (rho=.57, p<.001) and heart rate variability (rho=.70, p=.01).

Analysis of the Inter-Beat Intervals (IBIs) was performed using custom-built Matlab scripts. These scripts were designed through an extensive piloting process to be optimal for the ECG device used for this study. First, data were parsed using a simple amplitude threshold (see e.g. (Aurobinda, Mohanty, & Mohanty, 2016) for a similar approach), with R peaks identified as moments where the raw ECG signal exceeded the threshold value. Initially, the threshold value was set high; the same process was then repeated at incrementally decreasing thresholds.

At each threshold value, the R peaks identified were automatically subjected to the following checks. These threshold values were set following extensive piloting and visual inspection of our infant ECG data using the visualisation shown in Figure S2. i) minimum temporal threshold: does the R peak occur at a time interval of greater than 300 msecs since the previous R peak (corresponding to a heart rate of 200 BPM); ii) maximum temporal threshold: does the R peak occur at a time interval of less than 850 msecs since the previous R peak (corresponding to a heart rate of 70 BPM); iii) maximum rate of change: when we calculate the R to R interval between this peak and the subsequent peak, and compare it with the R to R interval between this peak and the previous peak, is this difference less than 300msecs? In setting these threshold values, careful attention was paid to visual inspection to determine the maximum and minimum ‘genuine’ heart rates observed in our infant data; in setting the maximum rate of change criterion, careful attention was paid to identify the maximum rate of vagally mediated heart rate changes in infants.

Figure S2 shows a sample screenshot from the Matlab processing algorithm that was used. Two separate types of artefact are shown. The first, highlighted by the call-out figures at a and d, are instances where the ECG signal for a particular beat was lower than the threshold, and a genuine beat was missed. It can be seen that in both instances, the R peaks either side of this missing beat have been automatically identified, and excluded. These artifacts were identified based on the maximum temporal threshold criterion in example a and d, and additionally based on the maximum rate of change criterion in example d. The second, highlighted by the call-out figures at b and c, are instances where the ECG signal exceeded the amplitude threshold, and an incorrect R peak was identified. In both instances, the incorrect beat has been identified based on the minimum temporal threshold criterion, and the R peaks either side of this incorrect beat have been identified and excluded. Please note also that the sample below has been selected in order to demonstrate how the program identified the most common artefacts in the data. Overall, the occurrence of both types of artefact in our data is relatively rare, as is shown in Figure S3, below.

These three criteria were applied separately to data after it had been parsed at each threshold value. Following this, at each threshold value, the proportion of candidate R peaks that were rejected was compared with the proportion of candidate R peaks that passed all three criteria. The threshold value with the lowest proportion of rejected candidate R peaks was chosen as the threshold used for that participant.

In addition, and as a further check, a trained coder who was naïve to study hypotheses double coded a randomly selected subsample of 1000 beats for 20% of the participants, coding them as genuine or artefactual. Cohen’s kappa was calculated to measure inter-rater reliability between the manual coding and the automatic coding, based on the best-fitting threshold level. This was found to be 0.97, which is high (McHugh, 2012).


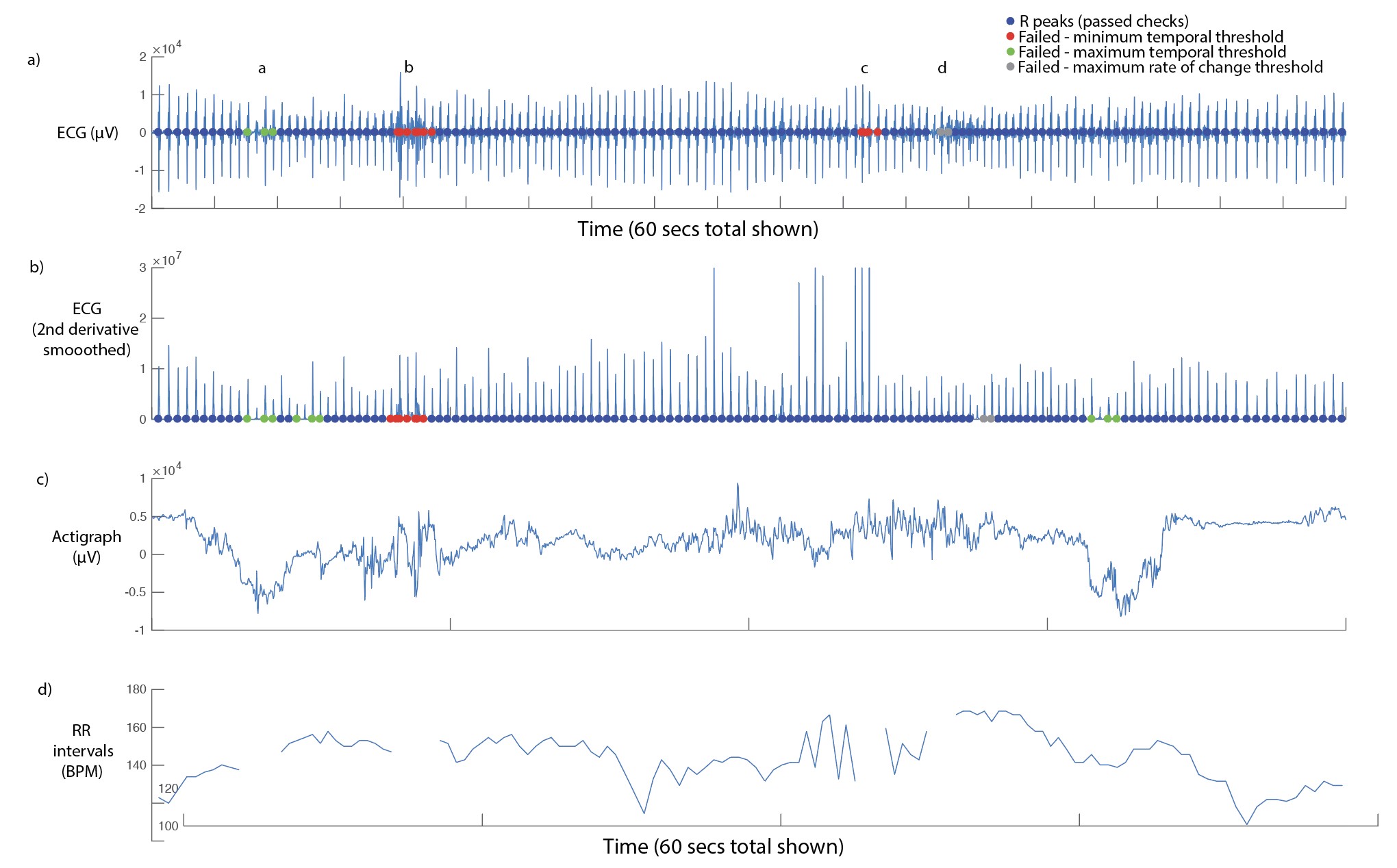


*Figure S2: Sample screenshot from ECG parsing algorithm. 60 seconds’ data is shown. From top to bottom: i) raw ECG signal. Coloured dots show the results of the three checks described in the main text, below (see legend); ii) smoothed second derivative of ECG signal. This measure was not used as our pilot analyses found it to be less effective than applying the processing to the raw signal; iii) raw (unprocessed) actigraph data. This information was only used for visual inspection, and was not used in parsing; iv) RR intervals (in BPM), with rejected data segments excluded.*

Figure S2 below shows a histogram of the proportion of candidate R peaks rejected for each participant, based on the best-fitting threshold value. The median (st. err.) is 1.07 (0.36) % data rejected. This relatively low figure was achieved through very close attention during the piloting phase to the selection and placement of the ECG electrodes, to the design of the device, and the gain settings on the recording device.


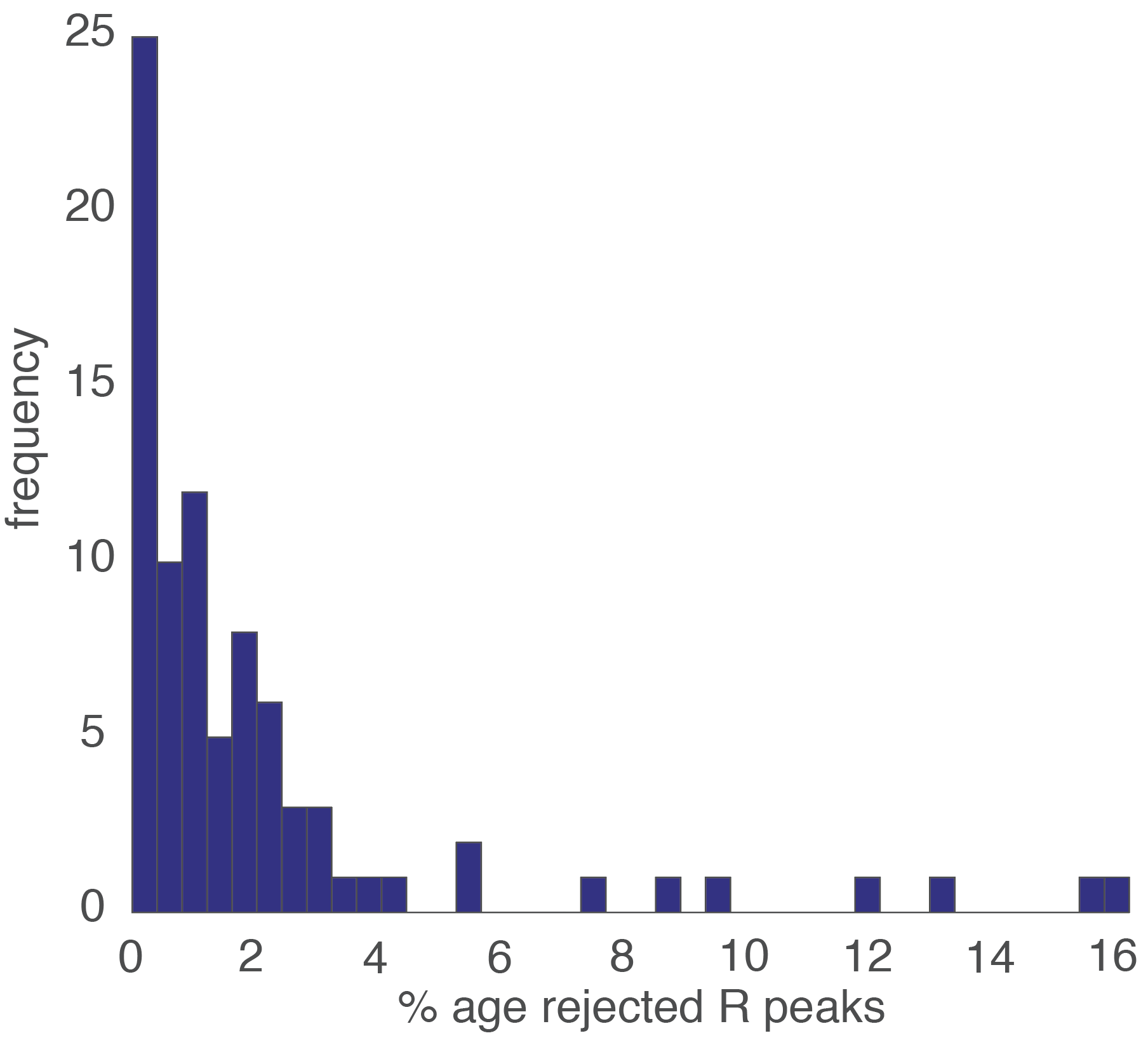


*Figure S3: Histogram showing the proportion of rejected R peaks (as identified using the three criteria described above).*

## 1.4 Heart-Rate Variability (HRV)

HRV was calculated using the PhysioNet Cardiovascular Signal Toolbox (Vest et al., 2018). In these scripts, which performed a completely separate analysis of the ECG data, a 60-second window with an increment of 60 seconds was implemented, and the default settings were used with the exception that the min/max inter-beat interval was set at 300/750 ms for the infant data and 300/1300 ms for the adult data. The Root Mean Square of Successive Differences (RMSSD) measure was taken to index Heart Rate Variability, but other frequency domain measures were additionally inspected and showed highly similar results, as expected (Vest et al., 2018).

###

## 1.5 Actigraphy

Actigraphy was recorded at 30Hz. To parse the actigraphy data we first manually inspected the data, then corrected artifacts specific to the recording device used, then applied a Butterworth low-pass filter with a cut-off of 0.1 Hz to remove high-frequency noise, and then averaged from three dimensions into one. Actigraphy data were available for all participants tested.

## Arousal composite

Previous research has shown significant patterns of tonic and phasic covariation between different autonomic measures collected from infants (Wass, Clackson, & de Barbaro, 2016; Wass, de Barbaro, & Clackson, 2015). Here, we include plots showing that the present dataset replicated and extended these results. The plots only show the sections of the data when participants were at home, comparing sections in which the infants were awake and asleep. Figure S2a shows cross-correlation plots examining the relationship between heart rate and movement. In both waking and sleeping sections the zero-lag correlation is 0.5. Figure S2c shows how these zero-lagged correlations vary on a per-participant basis. S2b shows an illustrative sample from a single participant. Sleeping sections show very low movement levels and lower heart rate. Of note, heart rate and movement do still inter-relate during the sleeping sections of the data (Figure S2c), albeit that the variability in heart rate and movement is lower. Figure S2 d)-f) show similar relationships between heart rate and heart rate variability, illustrating the strong and consistent negative relationships that were observed between these variables, as predicted.


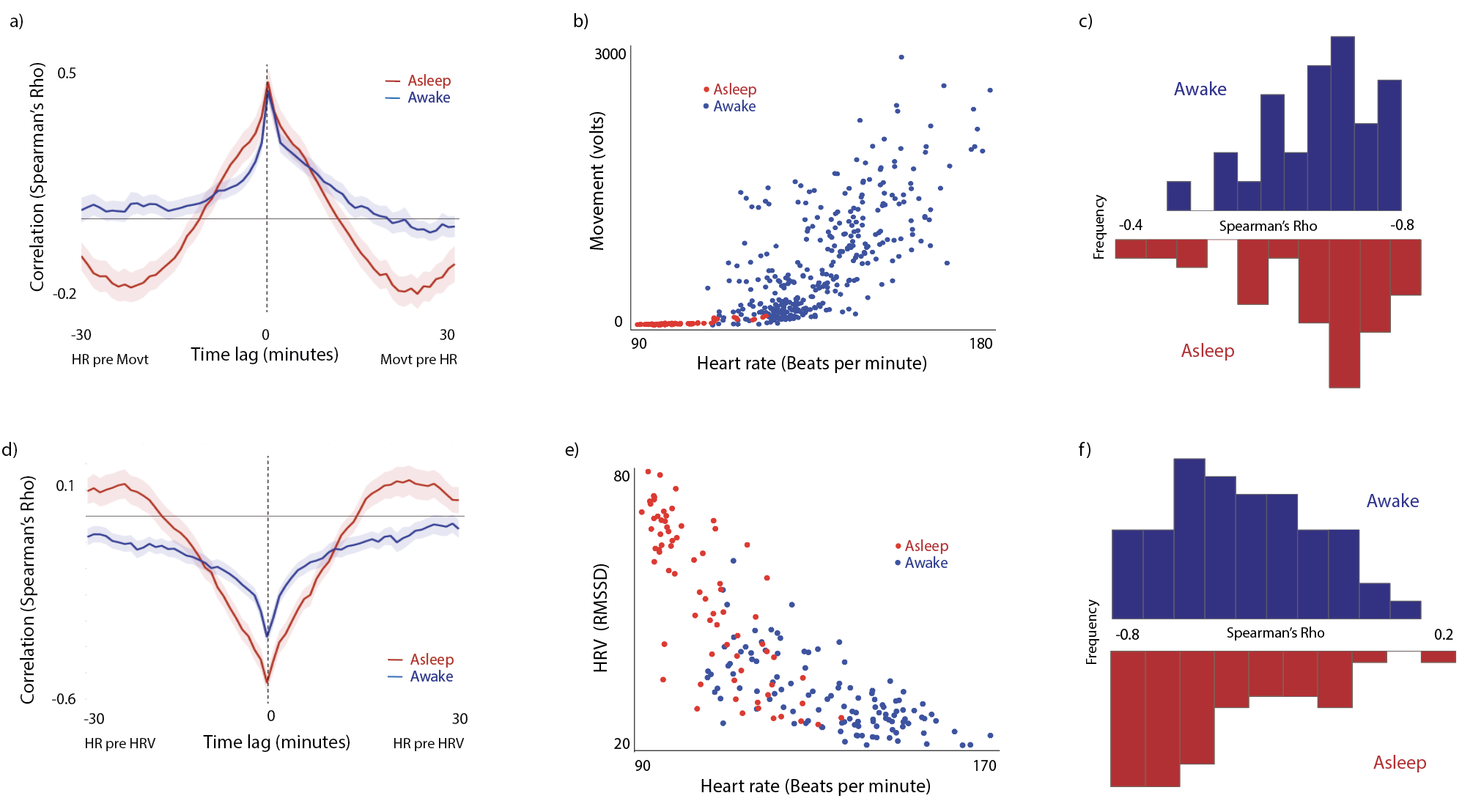


*Figure S4: Illustrating the relationship between the individual physiological measures included in the composite measure. a) Cross-correlation of the relationship between HR and Movement. b) Scatterplot from a sample participant. Each datapoint represents an individual 60-second epoch of data. c) Histograms showing the average zero-lagged correlation between 60-second epochs, calculated on a per-participant basis and then averaged. d)-f) Equivalent plots for Heart rate and Heart rate variability.*

Extensive previous research has identified fractionation, and differentiation, within our autonomic response systems (Janig & Habler, 2000; Kreibig, 2010; Lacey, 1967; Levenson, 2014; Quas et al., 2014) – suggesting, for example that the sympathetic and parasympathetic subdivisions operate, to an extent, in a non-additive manner (Samuels & Szabadi, 2008). Although indubitable, these findings should not be seen as rendering incorrect our treatment here of autonomic arousal as a one-dimensional construct. Like many other arguments concerned with general versus specific factors, the question is rather one of the relative proportions of variance that can be accounted for by a single common factor in comparison with the variance accounted for by the sum of specific factors (Graham & Jackson, 1970) (see also (Calderon, Kilinc, Maritan, Banavar, & Pfaff, 2016)).

As a result of these considerations, the three autonomic measures were collapsed into a single composite measure for Analysis 1. To do this, the actigraphy data was first subjected to a log transform (Thomas & Burr, 2008), to correct the raw results, which showed a strong positive skew (Wass et al., 2016; Wass et al., 2015) (see also SM section 1.6, below). Second, all three variables were converted to z-scores. Third, the HRV data were inversed because of the overall negative relationships noted between HRV and the other two measures (see Figure S4). Fourth, the three z-scores were averaged.

On the occasions where heart rate data were excluded due to artifact, data from actigraphy alone was used for the composite variable. Note that these occasions were relatively rare (accounting for a median ~=1% of all data - see Figure S3), and that the zero-lag cross-correlation between movement and heart rate across all available data was high (~=.5 – see Figure S4).

## 1.7 Removal of autocorrelation from arousal data

Autonomic arousal data are known to show autocorrelation (Wass et al., 2016). In order to preclude the possibility that differences in the autocorrelation may have influenced results, the autocorrelation was removed from the data prior to performing all calculations, using the following procedure. First, best-fit bivariate polynomials were calculated for the two time series independently, in order to remove linear and quadratic trends, and the residuals obtained were subjected to the Dickey-Fuller test to check that they showed stationarity, which they did. The residuals were used in subsequent analyses. Next, in order to remove the autocorrelation component from each time series independently, univariate autoregressive models were fitted to each time series, and the residuals were calculated (see e.g. Feldman, Greenbaum, & Yirmiya, 1999; Feldman, Magori-Cohen, Galili, Singer, & Louzoun, 2011; Jaffe et al., 2001; Suveg et al., 2016 for similar approaches). The residual values (shown in Figure 1) were converted into z-scored values. These z-scored values were then used for all analyses. The only exception to this is the analyses specifically examining changes in autocorrelation relative to vocalisations, for which the raw uncorrected data were used.

## Home/Awake coding

### 1.8.1 Home/not home

Coding of when participants were at home was performed using the GPS monitors built into the recording devices. The position of the participant’s home was calculated based on the postcode data that they supplied, and any GPS samples within a 50m area of that location were treated as Home (corresponding to the accuracy of the GPS devices that we were using).

### 1.8.2 Sleeping/waking

To identify samples in which infants were sleeping, parents were asked to fill in a logbook identifying the times of infants’ naps during the day. This information was manually verified by visually examining the actigraphy and ECG data collected, on a participant by participant basis. Actigraphy, in particular, shows marked differences between sleeping and waking samples (see Figure 1 in main text), which allowed us to verify the parental reports with a high degree of accuracy. N=4 of the participants recorded did not sleep during the day that we were recording.

## Supplementary analysis – simulation to examine the effects of sparse sampling on the data.


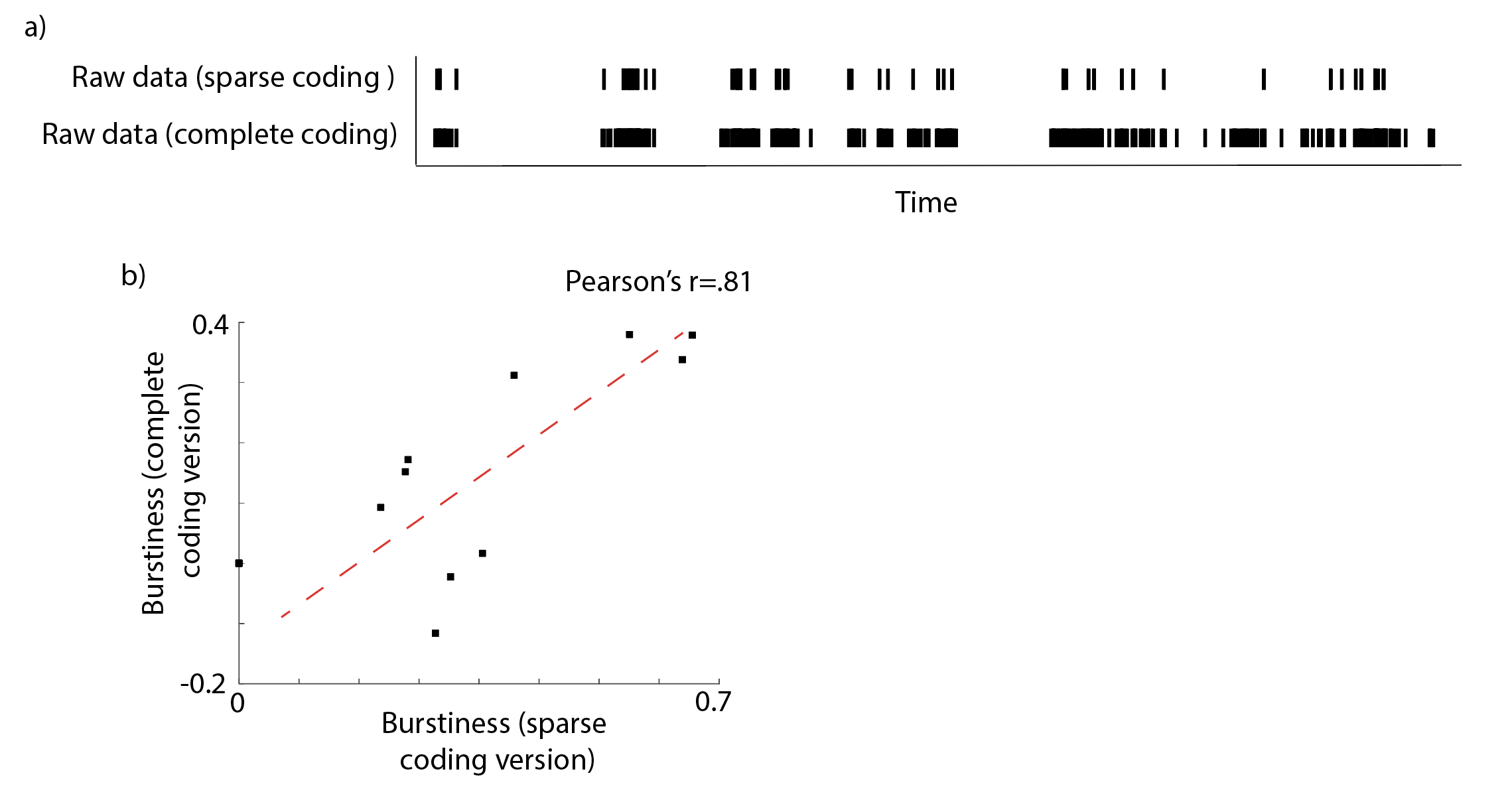


*Figure S5. Examination of how sparse sampling affected the temporal distribution of our data. a) Example raw data file comparing a two-hour long segment of fully coded data (containing all vocalisations recorded, based on continuous recording) with a ‘sparse coding’ simulation (containing just the vocalisations recording during the first 5 seconds of every minute). b) We obtained N=10 continuous hour-long recordings from 5-10-month-old infants and examined the temporal distribution of the data, comparing the continuous recording with the sparse coding simulation described in b). To quantify the temporal distribution of the data we calculated the burstiness (following the equation used in* ^68^*). Scatterplot shows the relationship between the burstiness as estimated from the complete coding version and from the sparse coding version. The Pearson’s r between the two measures was r(9)=.81, p<.001.*

# Supplementary Results

## 2.1 Part 2 – repeated with quartile split by household chaos


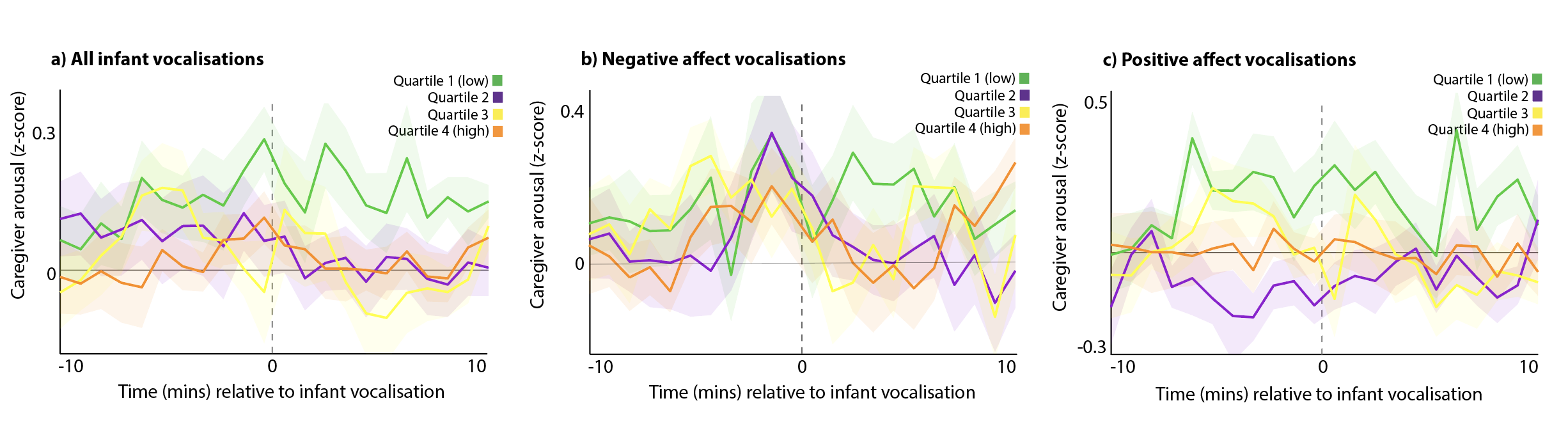


*Figure S6: Identical to Figure 3, but based on a quartile split by household chaos.*

## 2.2 Part 2 – repeated with median split by caregiver depression


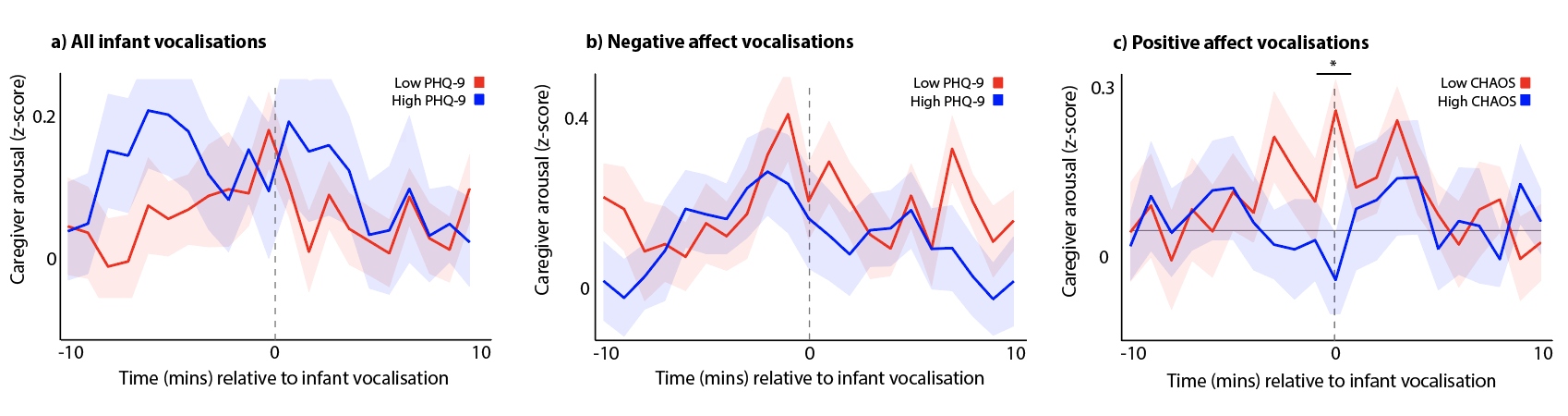


*Figure S7: Identical to Figure 3, but based on a median split by caregiver depression (measured using the PHQ-9). High PHQ-9 indicates elevated caregiver depression. * - sections identified as showing significant group differences by the permutation-based cluster analysis *<.05.*

## 2.2 Part 2 - Caregiver arousal changes around low- and high-intensity infant vocalisations


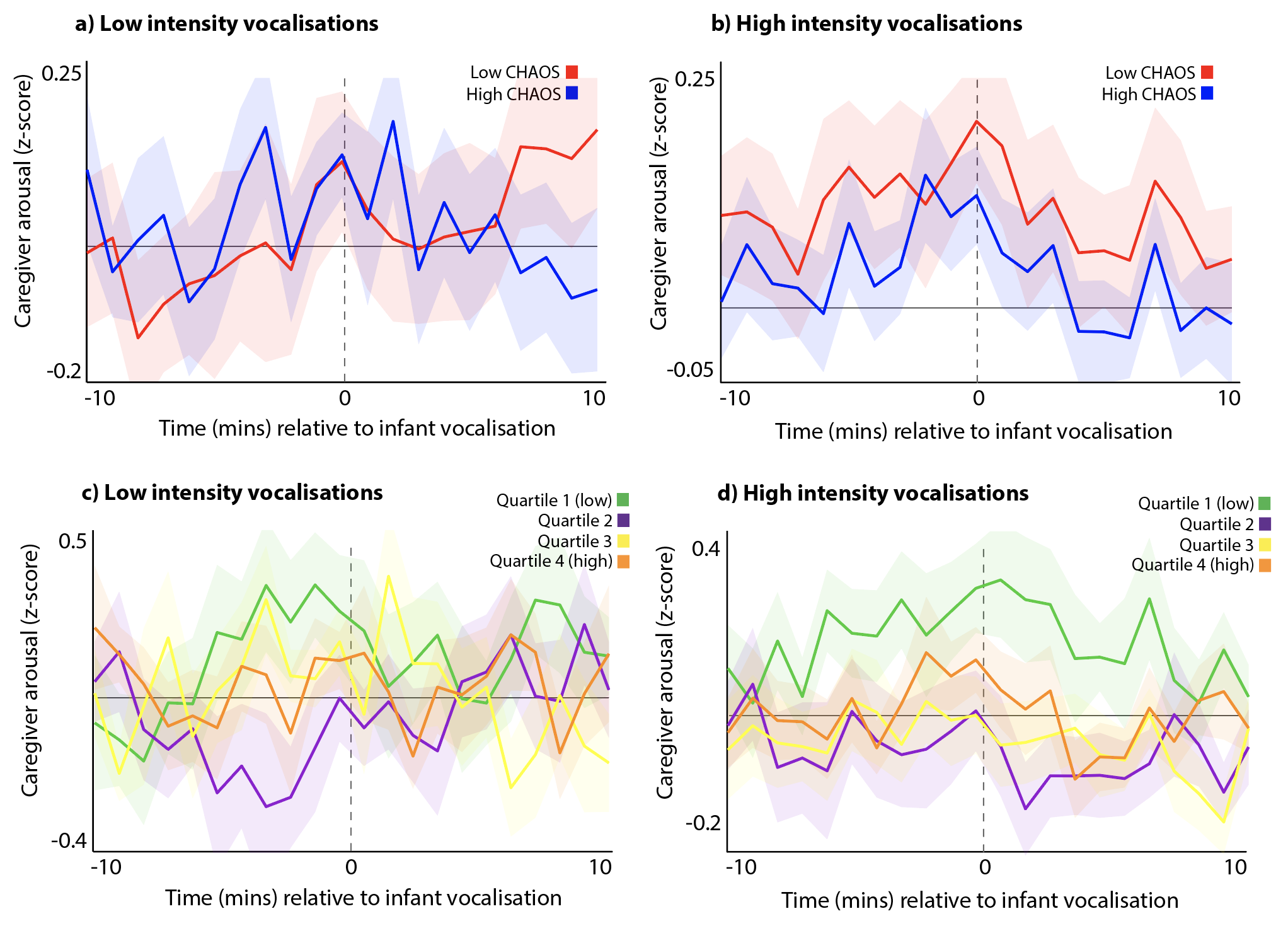


*Figure S8: Identical to Figure 4 but examining caregiver arousal changes to infant vocalisations subdivided by vocal intensity. a) and b) show results broken down using a median split by household chaos. c) and d) show the same analysis, but subdivided using a quartile split by household chaos.*

## 2.3 Part 3a – repeated with quartile split by household chaos


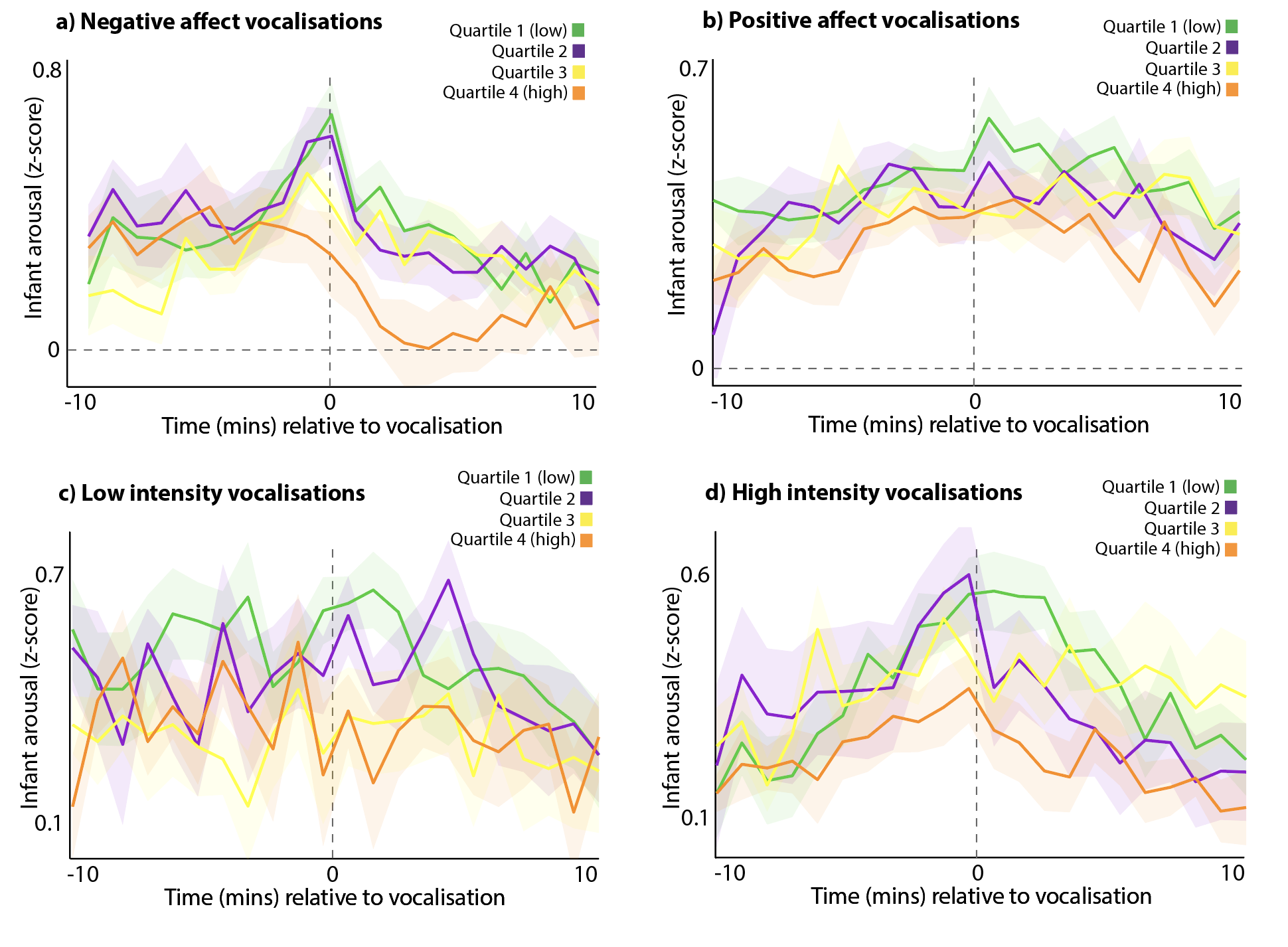


*Figure S9: Identical to Figure 5, but based on a quartile split by household chaos.*

## 2.4 Part 3b – repeated with quartile split by household chaos


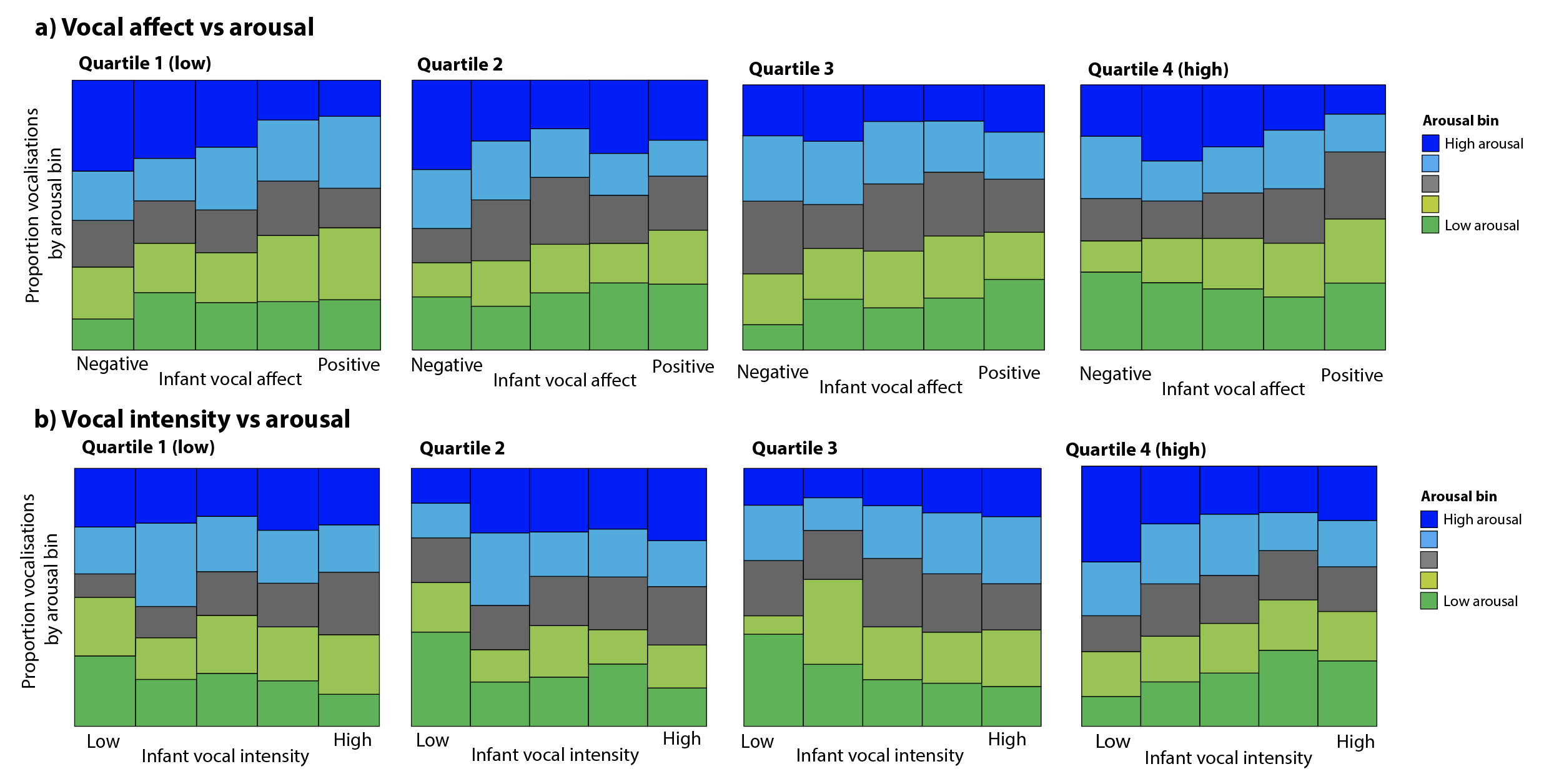


*Figure S10: Identical to Figure 6, but based on a quartile split by household chaos.*
